# RNA profiles reveal signatures of future health and disease in pregnancy

**DOI:** 10.1038/s41586-021-04249-w

**Published:** 2022-01-05

**Authors:** Morten Rasmussen, Mitsu Reddy, Rory Nolan, Joan Camunas-Soler, Arkady Khodursky, Nikolai M. Scheller, David E. Cantonwine, Line Engelbrechtsen, Jia Dai Mi, Arup Dutta, Tiffany Brundage, Farooq Siddiqui, Mainou Thao, Elaine P. S. Gee, Johnny La, Courtney Baruch-Gravett, Mark K. Santillan, Saikat Deb, Shaali M. Ame, Said M. Ali, Melanie Adkins, Mark A. DePristo, Manfred Lee, Eugeni Namsaraev, Dorte Jensen Gybel-Brask, Lillian Skibsted, James A. Litch, Donna A. Santillan, Sunil Sazawal, Rachel M. Tribe, James M. Roberts, Maneesh Jain, Estrid Høgdall, Claudia Holzman, Stephen R. Quake, Michal A. Elovitz, Thomas F. McElrath

**Affiliations:** 1 Mirvie, Inc., South San Francisco, CA USA; 2 Department of Epidemiology Research, Statens Serum Institut, Copenhagen, Denmark; 3 Brigham and Women’s Hospital, Boston, MA USA; 4 Department of Obstetrics and Gynecology, Herlev University Hospital, Herlev, Denmark; 5 Department of Women and Children’s Health, School of Life Course Sciences, Faculty of Life Sciences and Medicine, King’s College London, St Thomas’ Hospital Campus, London, UK; 6 Center for Public Health Kinetics, New Delhi, India; 7 Global Alliance to Prevent Prematurity and Stillbirth (GAPPS), Lynnwood, WA USA; 8 Department of Obstetrics & Gynecology, University of Iowa Hospitals & Clinics, Iowa City, IA USA; 9 Public Health Laboratory-Idc, Pemba, Zanzibar Tanzania; 10 Michigan State University, East Lansing, MI USA; 11 BigHat Biosciences, Inc., San Mateo, CA USA; 12 Department of Obstetrics, Zealand University Hospital, Roskilde, Denmark; 13 Department of Pathology, Herlev University Hospital, Herlev, Denmark; 14 Magee-Womens Research Institute, Department of Obstetrics and Gynecology and Reproductive Sciences, Epidemiology and Clinical and Translational Research University of Pittsburgh, Pittsburgh, PA USA; 15 Department of Bioengineering, Stanford University, Stanford, CA USA; 16 Chan Zuckerberg Biohub, Stanford, CA USA; 17 Department of Applied Physics, Stanford University, Stanford, CA USA; 18 Maternal and Child Health Research Program, Department of Obstetrics and Gynecology, University of Pennsylvania School of Medicine, Philadelphia, PA USA

**Keywords:** Gene expression, Predictive markers

## Abstract

Maternal morbidity and mortality continue to rise, and pre-eclampsia is a major driver of this burden^1^. Yet the ability to assess underlying pathophysiology before clinical presentation to enable identification of pregnancies at risk remains elusive. Here we demonstrate the ability of plasma cell-free RNA (cfRNA) to reveal patterns of normal pregnancy progression and determine the risk of developing pre-eclampsia months before clinical presentation. Our results centre on comprehensive transcriptome data from eight independent prospectively collected cohorts comprising 1,840 racially diverse pregnancies and retrospective analysis of 2,539 banked plasma samples. The pre-eclampsia data include 524 samples (72 cases and 452 non-cases) from two diverse independent cohorts collected 14.5 weeks (s.d., 4.5 weeks) before delivery. We show that cfRNA signatures from a single blood draw can track pregnancy progression at the placental, maternal and fetal levels and can robustly predict pre-eclampsia, with a sensitivity of 75% and a positive predictive value of 32.3% (s.d., 3%), which is superior to the state-of-the-art method^2^. cfRNA signatures of normal pregnancy progression and pre-eclampsia are independent of clinical factors, such as maternal age, body mass index and race, which cumulatively account for less than 1% of model variance. Further, the cfRNA signature for pre-eclampsia contains gene features linked to biological processes implicated in the underlying pathophysiology of pre-eclampsia.

## Abstract (web summary)

Expression signatures from cell-free RNA of pregnant women can be used to reveal normal biology of pregnancy and predict development of pre-eclampsia.

## Main

The period from conception to delivery represents the most rapid growth and development in an individual’s life. The ability to support this development requires dramatic and poorly understood alterations in maternal physiology. Research into human pregnancy has clear ethical constraints, and the unique character of human gestation has limited deeper understanding of the physiology and pathophysiology of pregnancy^3^. Haemochorial placentation is found among many mammalian species; however, in humans, it involves a unique degree of trophoblastic invasion^4,5^, and because pre-eclampsia occurs predominantly in humans, conventional animal models are of limited value^6,7^. Pre-eclampsia, a condition marked by maternal endothelial dysfunction and associated new-onset maternal hypertension, complicates up to 1 in 12 pregnancies and is a significant cause of maternal morbidity and higher lifetime risk of cardiovascular disease^1^.

Here we demonstrate the ability of cfRNA transcripts to establish the normative responses of both maternal and fetal tissues characteristic of normal pregnancy progression. By implication, deviation from normative cfRNA expression patterns should allow the prediction of impending pathology before its presentation. We demonstrate the use of cfRNA to characterize women at risk of pre-eclampsia months before diagnosis. Notably, the cfRNA profiles identify risk solely through molecular mechanisms common to pre-eclampsia and are therefore exclusive of clinical variables such as race, body mass index (BMI), maternal comorbidities and/or obstetrical history.

**Fig. 1:**
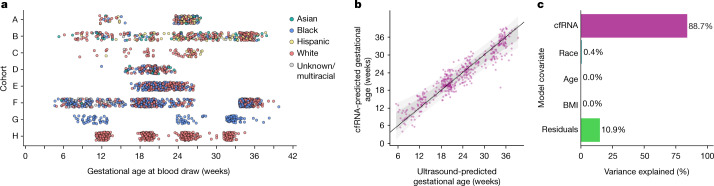
Overview of plasma sampling and cohorts and gestational age modelling. **a**, Cohorts are labelled A–H (Table 1). Circles represent plasma samples from liquid biopsies (*n* = 2,539). Colours represent the race of the maternal donor. **b**, Model predictions from the hold-out test (*n* = 474) using cfRNA transcript data in the Lasso linear model versus ultrasound-predicted gestational age. The dark grey zone represents 1 s.d., and the light grey zone represents 2 s.d. **c**, Variance explained from ANOVA.

In this study, we gather the largest and most diverse dataset of maternal transcriptomes to date. Samples were drawn from eight prospectively collected cohorts that provided *n* = 2,539 plasma samples from *n* = 1,840 pregnancies for women of multiple ethnicities, nationalities, geographic locations and socioeconomic contexts, while covering a range of gestational ages (Fig. 1a). The broad sociodemographic spectrum of our data (Table 1 and Supplementary Table 1) enabled us to test the applicability of maternal transcriptomes at one gestational time point. A detailed description of each cohort and the methodology is available in the Supplementary Information.

**Table 1:** Sample overview

Cohort	A	B	C	D	E	F	G	H
Blood draws (*n*)	201	385	69	186	353	793	140	412
Pregnancies (*n*)	197	219	68	186	352	592	120	106
% Asian	10.7	10.0	1.5	10.2	0.0	0.5	0.0	0.0
% Black	18.3	4.6	0.0	25.3	45.2	48.5	100.0	0.0
% Hispanic	24.4	17.8	14.7	0.0	0.0	0.0	0.0	0.0
% White	40.1	56.6	83.8	61.3	54.8	44.3	0.0	100.0
% Unknown or multiracial	6.6	11.0	0.0	3.2	0.0	6.8	0.0	0.0
Gestational age at blood draw (weeks)	12.0–27.9	5.6–38.2	8.9–28.1	12.2–23.8	16.9–26.8	4.9–40.2	8.0–38.7	11.4–34.8
BMI (kg m^−^^2^)^*^	28.1 ± 7.4	26.9 ± 6.2	33.3 ± 9.0	26.4 ± 6.2	28.6 ± 8.2	28.9 ± 7.6	24.5 ± 5.1	25.4 ± 6.1
Maternal age (years)^*^	32.4 ± 5.7	30.1 ± 5.1	29.8 ± 5.2	32.7 ± 5.4	26.5 ± 5.7	24.0 ± 4.5	28.8 ± 6.3	30.5 ± 4.7

^*^Variation shown as s.d.

Blood draw and pregnancy count, breakdown of ethnicity and race, and clinical factors.

## RNA signal independent of clinical factors

Ultrasound-based gestational age has long been used as a surrogate measure of pregnancy progression. Here, we show that a cfRNA signature is as accurate a measure of gestational age while also providing insights into the biology of pregnancy progression. As a first step to develop a machine learning model, we divided our data from all full-term pregnancies without complications into a training set (*n* = 1,908 samples) and a test set (*n* = 474 samples), stratified by gestational age so that all age strata were represented proportionally. Before modelling, we standardized the means of gene counts across all cohorts (Methods and Extended Data Fig. 5). A Lasso linear model was fitted to predict gestational age in the training set, with a test set performance of a mean absolute error of 14.7 days (Fig. 1b, Extended Data Fig. 6 and Supplementary Data 1), referencing to first-trimester fetal ultrasound biometry. Overall, the error of our model is equivalent to that of second-trimester ultrasound and superior to that with third-trimester ultrasound^8^, and could provide an alternative dating procedure for women who start prenatal care later in pregnancy.

Next, we explored whether inclusion of clinical variables altered model performance. By analysis of variance (ANOVA), we showed that the model was driven almost entirely by information from the cfRNA transcripts, with BMI, maternal age and race accounting for less than 1% of variance (Fig. 1c). Rebuilding the gestational age model including maternal race, BMI and age provided no improvement in accuracy (0.07 days, not significant by bootstrap test).

## Fetal signatures in maternal circulation

**Fig. 2:**
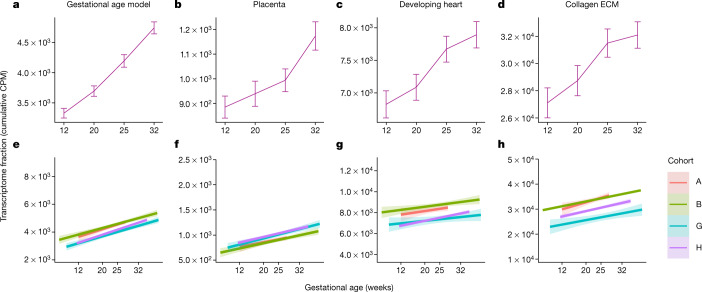
Temporal profiles of pregnancy pathways for gene sets from the gestational age model and independently identified gene sets for placenta, developing fetal heart and collagen extracellular matrix known to be involved in uterus and cervix growth over gestation. **a**–**d**, Maternal plasma transcriptome fractions for gene sets averaged across all samples in each collection window. Gestational age model (**a**), placenta (**b**), developing heart (**c**) and collagen extracellular matrix (ECM) (**d**). Error bars correspond to the 95% confidence interval around the mean. CPM, counts per million. *n* = 93 for each time point and gene set. **e**–**h**, Signal across all cohorts with longitudinal data: gestational age model (**e**), placenta (**f**), developing heart (**g**) and collagen ECM (**h**). Linear fits are shown of transcriptome fractions for all samples across corresponding gestational ages recorded at collection times. The band around the solid line corresponds to the 95% confidence interval. All slopes for the gestational age coefficients are distinct from 0 at a confidence level of 0.05. Cohort is indicated by colour.

**Extended Data Fig. 1:**
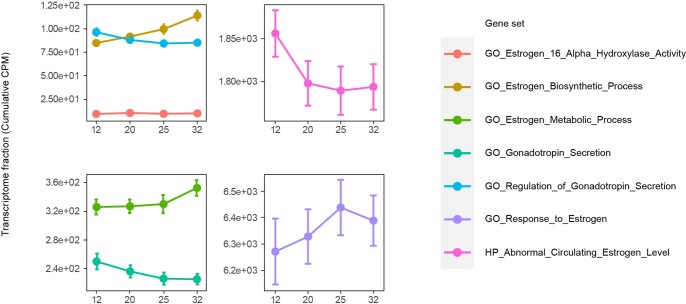
Temporal profiles of pregnancy-related endocrine signatures during pregnancy. Seven pregnancy-related gene ontology term signatures identified as highly significantly enriched (α=0.01) were profiled across collection times using cumulative CPM. Plasma transcriptome fractions for each gene set were averaged across all samples in each collection window with error bars corresponding to the 95% confidence interval around the mean. Panels correspond to different ranges of CPM, for the ease of comparison. CPM, counts per million. N=93 for each timepoint and gene set.

**Extended Data Fig. 2:**
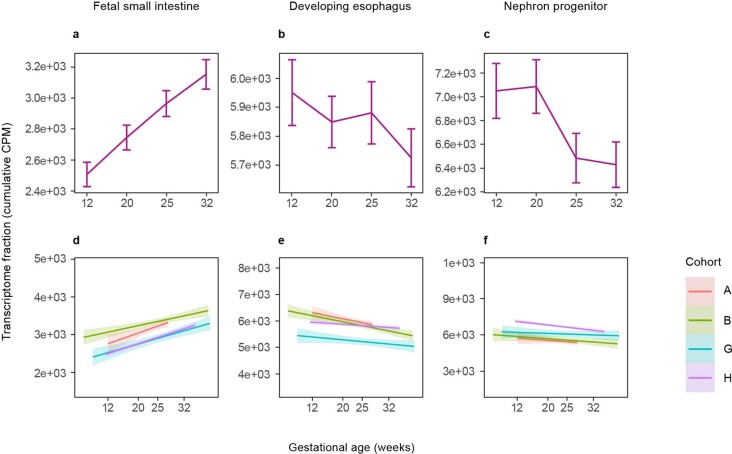
Temporal profiles of fetal gene sets from developing kidney and gastrointestinal tract. **a-c**, Maternal plasma transcriptome fractions for gene sets averaged across all samples in a given collection window. Error bars correspond to the 95% confidence interval around the mean. CPM, counts per million. N=93 for each timepoint and gene set. **d-f**, signal across all cohorts with longitudinal data. Linear fits of transcriptome fractions for all samples across corresponding gestational ages recorded at the collection times. The band around the solid line corresponds to the 95% CI. All slopes for the gestational age coefficient are distinct from 0 at a confidence level of 0.05. Cohort is indicated by color.

As the cfRNA signatures for gestational age demonstrated a dynamic change in transcripts as pregnancy progresses, we then explored whether transcripts found in the maternal circulation during pregnancy could be linked to their tissue of origin. Specifically, we sought to ascertain whether the molecular status of the placenta, fetal organs and/or maternal tissues (cervix and/or uterus) could be assessed by examining cfRNA profiles. While fetal cells are known to pass into the maternal circulation^9,10^, individual transcripts from the fetus or fetal cell types are relatively rare in maternal plasma; thus, we investigated these signals by analysing gene sets from Gene Ontology^11^ or the Molecular Signatures Database^12,13^. Using longitudinal data from cohort H covering 93 women sampled four times during pregnancy (Supplementary Information), we first confirmed that we could identify pregnancy-related sets such as those for gonadotropin and oestrogen pathways (Extended Data Fig. 1) and that the signal from the gestational age model increased with gestational age as did signal from the placenta (Fig. 2a, b and Methods). We show that hundreds of independently identified gene sets in maternal blood mirror the maternal and fetal physiological changes expected during pregnancy. Specifically, using single-cell RNA-seq data from adult and fetal organs (Supplementary Table 2), we were able to confirm changes in fetal gene sets, including those involved in fetal heart development, in maternal blood (Fig. 2c). Furthermore, the cfRNA profiles reflect expected changes in maternal tissues, such as the uterus and cervix, with progressively increasing expression of collagen and extracellular matrix gene sets^14^ (Fig. 2d). Extended Data Fig. 2 shows additional examples of fetal gene sets, including those of nephron progenitor cells for which expression become less abundant with gestational age in accordance with a decrease in the nephrogenic zone width^15,16^ and those in the gastrointestinal tract, where the oesophagus develops early with associated gene expression decreasing later versus small intestine where associated gene expression shows a steady increase^17^.

**Extended Data Fig. 3:**
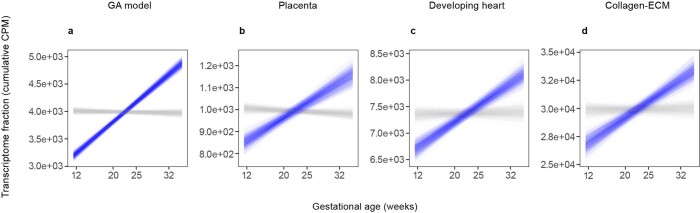
Bootstrapping with and without time-scrambling. **a-d**, for each of the significantly enriched gene sets, the trends were evaluated by bootstrapping (B=1,000) the original data (blue lines) and time-scrambled data (grey lines) obtained by reshuffling collection times.

To test whether the identified gene sets were uniquely associated with pregnancy progression, we next compared the observed gestational age collection time labels to a set of randomly permuted collection time labels. This comparison verified that all selected gene sets were associated with pregnancy progression (Extended Data Fig. 3). The directional signals could be confirmed in three independent cohorts (*n* = 351 women) for which longitudinal data were available (Fig. 2e–h). In all cases, the slopes for the gestational age coefficients were distinct from 0 at a 0.05 confidence level. In total, we tested 793 gene sets from single-cell analyses^12,13^, comprising 384 gene sets from adult and 409 gene sets from fetal tissues. Of these, 129 gene sets (55 fetal) were significantly correlated with gestational age, of which 99 gene sets (40 fetal) showed increased signal and 30 gene sets (15 fetal) showed decreased signal as a function of gestational age at collection in cohort H, and were confirmed in at least two other cohorts with longitudinally sampled individuals (Supplementary Data 2). As changes in these predefined gene sets were only significant in the context of gestational age across at least three cohorts with longitudinal information, we present here a non-invasive window into maternal–fetal development from a maternal blood sample.

## Early prediction of pre-eclampsia

Having established that cfRNA profiles can reveal and characterize molecular changes in the maternal–placental–fetal unit over gestation, it is likely that disruption of these pathways might identify women at risk for adverse pregnancy outcomes such as pre-eclampsia.

**Fig. 3:**
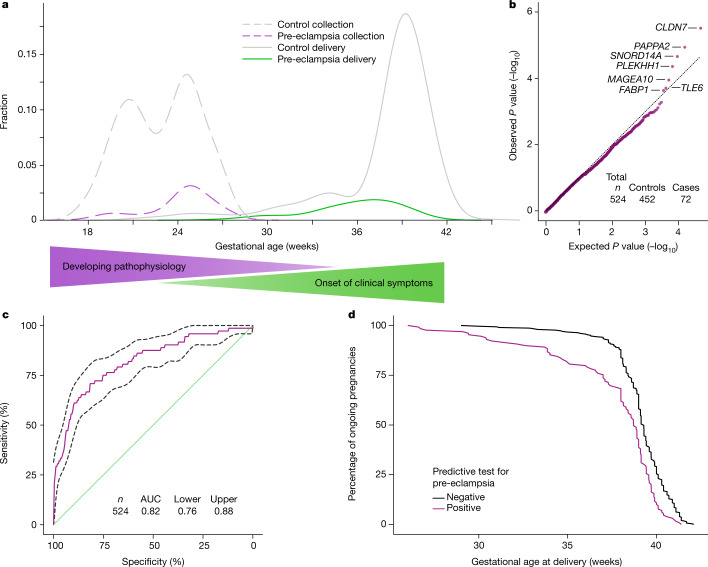
Features and model performance for prediction of pre-eclampsia. **a**, Sample collection time (dashed lines) and delivery time (solid lines) for women with pre-eclampsia (purple and green) and controls (grey). Gradients illustrate timelines for developing pathophysiology and onset of clinical symptoms. **b**, Quantile–quantile plot of ranked Spearman *P* values for women with pre-eclampsia (cases) versus controls. *P* values were calculated from Spearman correlation on cohort-corrected data for each gene. The genes used in the model are labelled. The black dotted line represents the expectation. **c**, Receiver operating characteristic curve (mean and 95% confidence interval) for the logistic regression model for pre-eclampsia (*n* = 524). **d**, Kaplan–Meier curves of deliveries in test-positive and test-negative populations (*n* = 439), excluding spontaneous preterm deliveries.

We evaluated the ability of cfRNA signatures in maternal blood, during the second trimester (16–27 weeks), to predict the development of pre-eclampsia. Maternal blood draws occurred, on average, 14.5 weeks (s.d., 4.5 weeks) before delivery (Fig. 3a); in contrast to work by Munchel et al.^18^ where plasma was collected at the time of diagnosis, the gestational age time points in our analysis correspond to timepoints where women are asymptomatic. A case–control study with 72 cases of pre-eclampsia and 452 non-cases selected from two independent cohorts (cohorts A and E) was performed (Supplementary Information). Cohort E included 31 controls with chronic hypertension and 19 controls with gestational hypertension and both cohorts included spontaneous preterm birth samples along with the normotensive term controls. Pre-eclampsia was defined by criteria consistent with those from the 2013 Task Force on Hypertension in Pregnancy (ACOG 2013), and each case was adjudicated by two board-certified physicians. As before, a cohort correction was applied before modelling.

Two-sided Spearman correlation tests identified signatures that separated the cases and controls; in each round of cross-validation, we retained features with an adjusted *P* value below 0.05 (Methods) and consistently identified seven genes: *CLDN7*, *PAPPA2*, *SNORD14A*, *PLEKHH1*, *MAGEA10*, *TLE6* and *FABP1* (Fig. 3b).

Four of the genes selected for modelling have functions relevant to pre-eclampsia or placental development. *PAPPA2*, encoding pregnancy-associated plasma protein 2, is expressed in the placenta^19^, specifically in trophoblast cells. It has previously been linked to the development of pre-eclampsia and has been associated with inhibition of trophoblast migration, invasion and tube formation^20,21^. Claudin 7 (*CLDN7*) is involved in tight cell junction formation and blastocyst implantation; in healthy pregnancies, expression of *CLDN7* is reduced in response to oestrogen at the time of implantation^22,23^. Similarly, *TLE6* has also been linked to preimplantation and early embryonic lethality^24^. Fatty acid-binding protein 1 (*FABP1*) was first purified from human cytotrophoblasts and is known to be highly expressed in the fetal liver; it is critical for fatty acid uptake and transport^25^ and is upregulated threefold when cytotrophoblasts differentiate to syncytiotrophoblasts at implantation^26^. The other three genes that make up the pre-eclampsia cfRNA signature (*SNORD14A*, *PLEKHH1* and *MAGEA10*) have been associated with pre-eclampsia through bioinformatic analyses, although their function is less well understood^27,28^. Two of the identified genes, *PAPPA2* and *FABP1*, were also identified in the gestational age model and highlight the imbalance in cfRNA signatures between pregnancy progression and pathology.

On the basis of these identified gene features, a logistic regression model in a leave-one-out cross-validation set-up was used to estimate the probability of pre-eclampsia. This model framework was chosen on the basis of learning curve analyses (Methods and Extended Data Fig. 7). At a sensitivity of 75%, our cfRNA model achieved a positive predictive value (PPV) of 32.3% (s.d., 3%) given a prevalence of pre-eclampsia of 13.7% in our study, superior to PPVs reported from current clinical state-of-the-art models, which are driven largely by maternal factors^2^ ; the area under the curve (AUC) for the model was 0.82 (95% confidence interval, ±0.06; Fig. 3c). Consistent with our findings with the gestational age model, inclusion of clinical variables (maternal BMI, age and race) had no effect on performance, as the classifier assigns zero weight to these clinical variables and they explain <1% of the variance based on ANOVA analyses. The lack of contribution to cfRNA profiles from clinical factors highlights the generalizability of these profiles to diverse populations.

When comparing gestational age at delivery between test-positive and test-negative individuals, a significant shift was found in the timing of delivery, with the test-positive population delivering earlier during gestation (*P* < 2 × 10^–7^; Fig. 3d). A positive test correctly identified 73% of individuals destined to have a medically indicated preterm birth over 3 months in advance of the onset of clinical symptoms or delivery.

**Extended Data Fig. 4:**
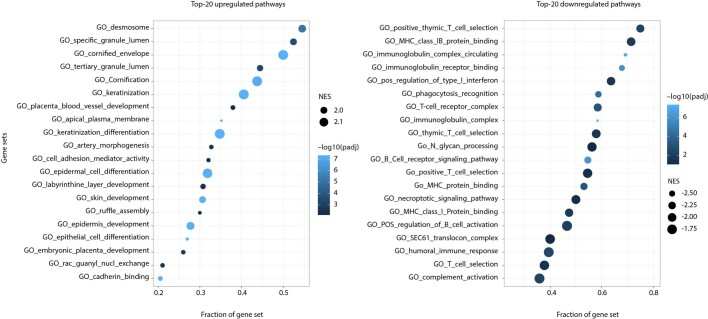
Gene set enrichment analysis of preeclampsia for gene ontology (GO) gene sets. **a**, Top-20 significantly upregulated gene sets. **b**, Top-20 significantly downregulated gene sets. Color gradient for adjusted *p*-value. NES, absolute normalized enrichment score.

To further understand molecular signature changes and how they might reflect the pathophysiology driving pre-eclampsia, we performed pathway analysis. The top upregulated pathways were dominated by structural cell functions, including placental blood vessel development, artery morphogenesis and embryonic placental development (Extended Data Fig. 4a), while the majority of downregulated pathways were related to immune pathways (Extended Data Fig. 4b). Both the upregulated and downregulated gene sets aligned with the accepted mechanism of pathogenesis for pre-eclampsia^29^.

In cohort E, the non-case group contained both normotensive women (*n* = 263) and women with chronic (*n* = 31) or gestational (*n* = 19) hypertension. Genes identified through comparison of the groups with chronic or gestational hypertension with the normotensive group showed no overlap with genes significant for pre-eclampsia (two-sided Spearman correlation test, *P* < 0.05). Additionally, no genes were differentially expressed in the chronic or gestational hypertensive groups when compared with the normotensive group. While others have published studies designed to determine the effect of hypertension more generally on gene expression (e.g., Zeller et al.^30^), here, we demonstrate that the signal for pre-eclampsia is specific to hypertension driven by a placental disorder and the signature is independent of signals associated with chronic hypertension. Clinically, it can be quite challenging to differentiate superimposed pre-eclampsia in women with pre-existing hypertension from exacerbation of baseline chronic hypertension. This difference is important, as one requires delivery for cure while the other usually does not.

As pre-eclampsia and spontaneous preterm birth are theorized to have some overlapping molecular pathways^31,32^, we tested whether excluding non-case samples with deliveries before gestational week 37 (*n* = 85) would affect test prediction. Removal of spontaneous preterm delivery samples did not alter the performance of the model (AUC = 0.79; 95% confidence interval, ±0.06), suggesting that inclusion of spontaneous preterm birth samples in the non-case group does not affect the pre-eclampsia classifier.

We report a standalone molecular predictor that has the potential to be an early detector of pre-eclampsia with a PPV of 32% that is based entirely on transcripts and is exclusive of clinical variables. This predictor contrasts with state-of-the-art methods, which are dependent on clinical factors and achieve a PPV of 4.4%^2^.

## Discussion

While other studies have looked at circulating biomarkers, a recent comprehensive review^33^ concluded that more data early in pregnancy are needed to support clinical value. Here, we reveal the ability of cfRNA transcripts to provide comprehensive molecular profiles of pregnancy progression by including signals from the placenta and the fetus. We have shown that novel transcript signatures from a single blood sample can (1) accurately track pregnancy progression independently of clinical factors and (2) reliably identify women at risk of developing pre-eclampsia months before presentation of the disease. Given the large sample size and diversity in our study population, it is noteworthy that race has a negligible effect on the expression patterns of gestational age estimates and pre-eclampsia risk evaluation. These findings allow for the development of personalized assessments for pregnancy.

Equally important, our work allows for the assessment of maternal risk independently of clinical factors, such as race, that are fraught with bias. The inclusion of race in clinical assessments results in miscalculation of patient risk and underdiagnoses^34–36^. While we acknowledge that, within specific subpopulations, the prevalence of complications such as pre-eclampsia may be higher, the evaluation of cfRNA transcripts directly exposes the developing pathophysiology. Further research will be needed to identify drivers of the identified pathophysiological pathways; the focus on molecular mechanisms allows stratification of risk without the need for enrichment of ‘pretest’ probabilities based on maternal sociodemographic characteristics. Further, an understanding of the maternal–fetal–placental transcriptome also represents a vehicle by which comprehension of the biological underpinnings of maternal–fetal development can be improved and provides novel insights into interactions across the maternal–fetal dyad. This holds the promise of precision therapeutic interventions that can target molecular subtypes of pre-eclampsia and preterm birth.

Improvement in maternal outcomes has been limited by the inability to access pregnancy tissues and a lack of understanding of the specific molecular phenotypes that identify those at risk before onset of symptoms. Our findings can now be leveraged to more accurately provide information on future maternal and fetal health and disease. Thus, our approach opens new therapeutic windows to effectively decrease maternal and neonatal morbidity and mortality.

## Methods

### The Mirvie RNA technology

#### cfRNA isolation

Plasma samples received on dry ice from our collaborators were stored at –80 °C until further processing. Total circulating nucleic acid was extracted from plasma ranging in volume from ~215 µl to 1 ml, using a column-based commercially available extraction kit, following the manufacturer’s instructions (Plasma/Serum Circulating and Exosomal RNA purification kit, Norgen, 42800).

Following extraction, cfDNA was digested using Baseline-ZERO DNase (Epicentre) and the remaining cfRNA was purified using an RNA Clean and Concentrator-5 kit (Zymo, R1016) or an RNeasy MinElute Cleanup kit (Qiagen, 74204).

#### RT–qPCR assay

We performed PCR with reverse transcription (RT–qPCR) analysis to assess the relative amount of cfRNA extracted from each sample. We measured and compared the threshold cycle (*C*_t_) values from each RNA sample using a three-colour multiplex qPCR assay from the TaqPath 1-Step Multiplex Master Mix kit (ThermoFisher Scientific, A28526) and a Quant Studio 5 system. We also measured the *C*_t_ values for an endogenous housekeeping gene (*ACTB*; ThermoFisher Scientific, 4351368).

#### cfRNA library preparation

cfRNA libraries were prepared using the SMARTer Stranded Total RNAseq-Pico Input Mammalian kit (Takara, 634418) following the manufacturer’s instructions, except that we did not use ribo depletion. Library quality was assessed by RT–qPCR following the method described for assessing RNA measurements and fragment analysis on a Fragment Analyzer 5300 (Agilent Technologies).

#### Enrichment and sequencing

Libraries were normalized before pooling for target capture. We used a SureSelect Target Enrichment kit (Agilent Technologies, 5190-8645) and followed the manufacturer’s instructions for hybrid capture. Samples were quantified, and 50-bp, paired-end sequencing was performed on a Novaseq S2. Between 96 and 144 samples were pooled and sequenced per sequencing run.

#### Analysis for outliers

qPCR of *ACTB* as well as MultiQC sequencing metrics were monitored to eliminate sample outliers before performing gene expression analyses. Individual samples more than 3 s.d. from the mean were removed as outliers. A total of 193 of 2,732 samples (7.1%) were removed following this filtering.

#### Read processing

Reads were processed following a similar protocol to that reported in Ngo et al.^37^. Briefly, raw sequencing reads were trimmed using trimmomatic^38^ and then mapped to hg38 using the STAR aligner^39^. After removing duplicates using Picard tools, gene counts were generated with htseq^40^.

### Cohort correction and feature normalization

**Extended Data Figure 5:**
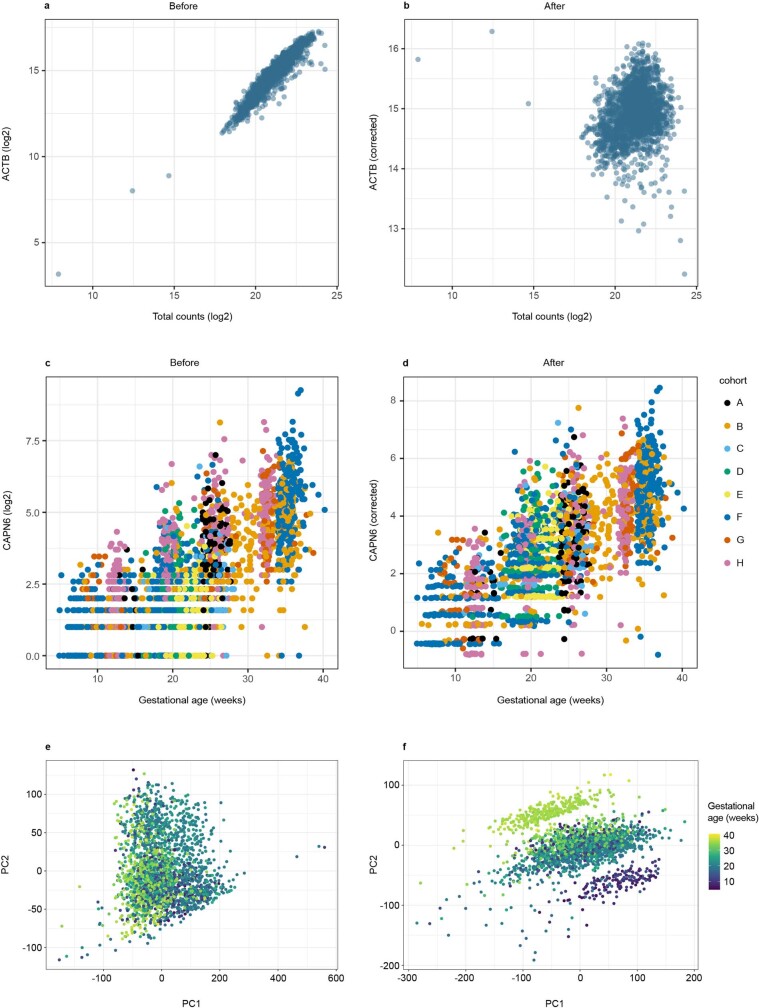
Effect of correcting for total count and cohort. Counts for ACTB as a function of total counts for the sample before (**a**) and after (**b**) correction. Counts for CAPN6 as a function of gestational age for all samples used in the gestational age model before (**c**) and after (**d**) cohort correction. Plot of first two principal components before (**e**) and after (**f**) cohort correction.

For each gene, its relationship to total counts per sample was measured and corrected using linear model residuals. Extended Data Fig. 5a, b shows what this looks like for the gene *ACTB*.

We also sought to correct the genes such that each cohort had the same mean value for each gene. However, the cohorts came from different parts of the gestational age spectrum. Therefore, only cohort effects orthogonal to the gestational age effect were corrected. This is shown in Extended Data Fig. 5c, d for the gene *CAPN6*. Each cohort was given its own colour.

Cohort E (bright yellow) had unusually low counts for its gestational age range before correction, and this effect was removed by correction.

Using principal-component analysis (PCA) to compress the high-dimensional space of all genes, the correction could be seen to clarify the separation of samples by gestational age as indicated by the colour gradient (Extended Data Fig. 5e, f).

### Linear correction algorithm

1. In the training, correct for (remove the effect of) the variable(s) of interest (e.g., gestational age) using linear model residuals.

2. Learn the required correction for the variables you wish to correct for in this corrected training data.

3. The residuals of that model (in the raw training and testing data) are your corrected data.

Note: the correction was learned entirely in the training data and the variable of interest in the testing data was never used, negating the possibility of a data leak.

### Lasso linear model for gestational age prediction and ANOVA

The Lasso model used in the gestational age model had its parameters chosen via 10-fold cross-validation in the training set. The largest cross-validation score within one standard error of the best cross-validation score was chosen (Breiman strategy). We limited our feature space by excluding pseudogenes and non-coding genes, as well as genes with median expression greater than zero, leaving a total of 13,208 features to evaluate. A final Lasso with this was then trained on the whole training set and evaluated in the test set. This was all done with the glmnet R package using the cv.glmnet() function.

**Extended Data Fig. 6:**
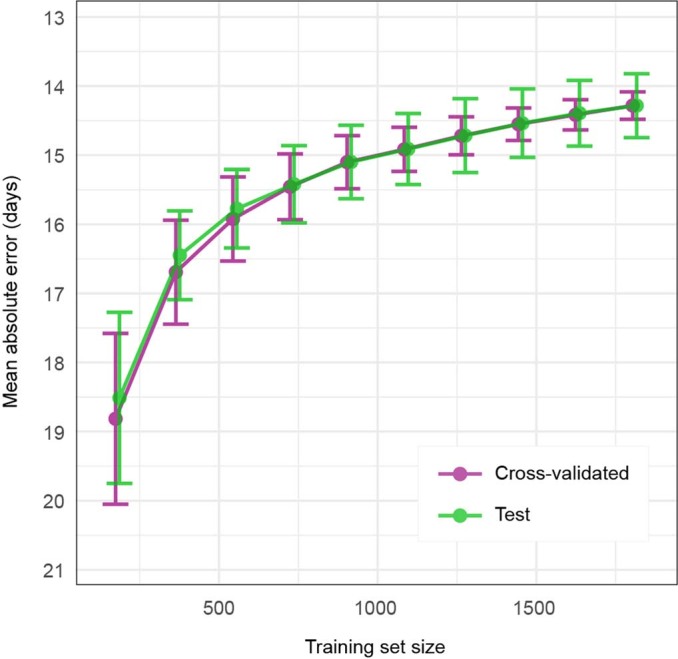
Learning curve for gestational age model. Model for gestational age is trained with increasing sample size, error is plotted for both training set (Cross-validated, purple) and held-out test set (green). Error bars are 1 standard deviation.

The model uses 674 of the available gene features (Supplementary Data 1), although this includes a long tail of features with low contribution. We tested performance for the 50 most informative features from the model and obtained a mean absolute error of 15.4 days. The continued reduction in error as we reached our complete training set of *n* = 1,908 samples indicated that model learning was not exhausted and that additional samples would have increased performance (Extended Data Fig. 6). Notably, as seen in Extended Data Fig. 6, the similar performance in cross-validation and on the independent held-out test data indicated that the model was not overfit with the 674 gene features. To determine how far the model could be extrapolated, a final model was built using all data; this gave a mean absolute error of 13 days across the entire dataset.

### Gestational age learning curve

The main gestational age modelling was done with an 80/20 train/test split. To assess model performance after decreasing amounts of training data, one can repeat analyses with 70/30 splits, 60/40 splits and so on (doing so repeatedly with different random splits to quantify uncertainty). In this way, one builds a learning curve (Extended Data Fig. 6) with different training set sizes on the *x* axis and model performance on the *y* axis.

### Gestational age model without cohort correction

For this approach, we selected all samples from healthy pregnancies and split the dataset into a training set (80% of data) and a test set (20% of data), in which samples were stratified by cohort. Samples that did not pass quality-control filtering based on basic sequencing metrics had been previously excluded from analysis. We trained a Lasso model to predict the gestational age at collection for each sample using the mean absolute error as an optimization metric and 10-fold cross-validation in the training set. We used all genes with mean log_2_(counts per million (CPM)  + 1)  > 1 (12,921 genes) plus a set of sequencing metrics as features for training. Modelling was performed in log_2_(CPM + 1) space, and all data were centred and scaled before modelling using the training set statistics. This led to a model with a mean absolute error of 15.9 days in the withheld test set using 487 transcriptomic features. We then selected the top 53 features of this model and retrained the Lasso using the same approach described above, achieving a mean absolute error of 16.6 days in the held-out test set.

### Gene set enrichment analysis

Gene set enrichment analysis (GSEA)^11,41^ was done with the fast GSEA algorithm^42^ using Bioconductor’s fgsea package^43^. Gene sets were compiled from the Molecular Signatures Database (MSigDB)^11,12^ using the CRAN msigdbr v7.2 API and directly from c8.all.v7.3.symbols.gmt. We focused on two collections of gene sets: the Gene Ontology (GO) subcollection of the ontology gene sets, C5:GO, and the cell type signature gene sets, C8 v7.3. Genes were ranked on the basis of their shrunken log-transformed fold change values and associated Wald test *P* values obtained from analysis of differential expression using Bioconductor’s DESeq2 (ref. ^44^), represented as –log_10_(*P* value)  × shrunkenLFC. GSEA was carried out on 372 samples from cohort H collected from 93 women with healthy pregnancies over four draw intervals during pregnancy, 11.4−14 weeks, 18−21 weeks, 22.8−27.8 weeks and 29.2–34.8 weeks. Shrunken log-transformed fold change values and corresponding *P* values were obtained from all six pairwise contrasts between the four draws. We used 102 fetal gene sets that were significantly enriched (Benjamini–Hochberg adjusted *P* < 0.01) in at least one pairwise comparison (Supplementary Table 2) in downstream analyses, including analysis of plasma transcriptome partitioning and set-specific longitudinal trends.

Using a GO collection of gene sets, we validated our approach and identified seven pregnancy-related sets that were significantly enriched in the comparison between early- and late-pregnancy samples (Extended Data Figure 1). Three gene sets in the gonadotropin and oestrogen pathways exhibited significant changes consistent with known physiology^45^.

### Evaluating changes in plasma transcriptome partitioning

The plasma transcriptome can be phenomenologically viewed as being partitioned into characteristic sets of genes. We assessed this partitioning in each cfRNA sample by converting raw gene counts to CPM and summing CPM over all genes in each of the sets. The resulting cumulative CPM score, which is a relative measure of the abundance of each gene set in the overall transcriptome, was used to directly compare gene sets across collection time points. Cumulative CPM scores for all gene sets significantly enriched between collections 1 and 4 were calculated for every cfRNA sample. The scores for each sample were regressed onto the recorded gestational age (in weeks) using a linear model. Gene sets with an adjusted *P* value for the gestational age coefficient <0.01 were considered as having a significant (positive or negative) trend in their relative abundance. The association of these trends with the time component in the data was further verified by scrambling the temporal structure and re-examining the trends along the original time variable. For each mother, we also evaluated the monotonicity of the cumulative CPM score function along the collection times. Because there are 24 possible permutations of order for the four collection times and only one of those permutations allows for a monotonic upward trend (with one for a downward trend), we were able to analytically assess the significance of the observed number of monotonic trends among 93 mothers using a chi-squared test.

### Pre-eclampsia analysis and learning curve

CIs for AUCs and sensitivity, specificity and PPV were all found via bootstrapping. PPV was calculated as PPV = (sensitivity × prevalence)/((sensitivity × prevalence) + ((1 – specificity) × (1 – prevalence))).

**Extended Data Fig. 7:**
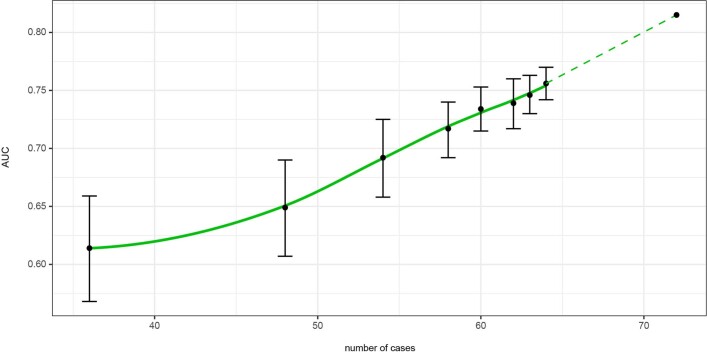
Learning curve for preeclampsia model. Model performance as a function of training set size. Error bars are 1 standard deviation.

To build the learning curve (Extended Data Fig. 7), we increased the size of the training set going from two- to ninefold cross-validation with a constant model: logistic regression with gene features chosen by Spearman correlation tests with an adjusted *P*-value threshold of 0.05. The point on the right connected to the learning curve via a dashed line is the leave-one-out cross-validation result shown in the main text.

### Reporting summary

Further information on research design is available in the Nature Research Reporting Summary linked to this paper.

## Online content

Any methods, additional references, Nature Research reporting summaries, source data, extended data, supplementary information, acknowledgements, peer review information; details of author contributions and competing interests; and statements of data and code availability are available at 10.1038/s41586-021-04249-w.

## Supplementary information

Supplementary Information This file contains a cohort overview, supplementary analyses, additional references and Supplementary Tables 1 and 2.

Reporting Summary

Peer Review Information

Supplementary Data 1 Full list of features selected by the Lasso for the gestational age model, including their weights in the model.

Supplementary Data 2 Full list of gene sets with significant negative correlation to gestational age in at least three independent cohorts. Gene sets were discovered in cohort H and confirmed in at least two other cohorts (A, B or G). The slope is the average change in gene set expression in CPM per week. Adjusted *P* value for discovery cohort. Number of cohorts with significant signal, including discovery cohort. Type, origin of dataset, fetal or adult tissue. Additional sheets for component genes for each set of adult or foetal genes.

Supplementary Data 3 Full list of gene sets with significant positive correlation to gestational age in at least three independent cohorts. Gene sets were discovered in cohort H and confirmed in at least two other cohorts (A, B or G). The slope is the average change in gene set expression in CPM per week. Adjusted *P* value for discovery cohort. Number of cohorts with significant signal, including discovery cohort. Type, origin of dataset, fetal or adult tissue. Additional sheets for component genes for each set of adult or fetal genes.

## Data Availability

Data are available with a signed data use agreement to protect identifiable data; please contact research@mirvie.com.

## Code availability

Code is available as three packages in the following repositories: mirmisc, 10.5281/zenodo.5604683; mirmodels, 10.5281/zenodo.5593282; and mirr, 10.5281/zenodo.5593280.
