# Supplementary material for: RNA profiles reveal signatures of future health and disease in pregnancy
Source: Nature. 2022 Jan 5;601(7893):422–7. doi: 10.1038/s41586-021-04249-w (PMC8770117; doi:10.1038/s41586-021-04249-w)
Supplement: Supplementary file 1 — This file contains a cohort overview, supplementary analyses, additional references and Supplementary Tables 1 and 2. [file 41586_2021_4249_MOESM1_ESM.pdf]

nature portfolio

https://doi.org/10.1038/s41586-021-04249-w

Supplementary information

RNA profiles reveal signatures of future health and disease in pregnancy

In the format provided by the authors and unedited

Supplementary Information.

Table of Contents

Cohort overview 2
Labels used to describe race and ethnicity 2
Cohort descriptions 2
Cohort A[47] 2
Cohort B 2
Cohort C[48] 3
Cohort D[49] 3
Cohort E[50] 4
Cohort F[51] 4
Cohort G[52] 4
Cohort H[53] 5
Supplementary analyses 5
Gestational age ANOVA 5
Table S3. ANOVA for gestational age. 5
Gestational age model without cohort correction 5
Pairwise enriched gene sets in cohort H 5
Table S4. Differentially expressed gene sets in all pairwise comparisons between blood draws in cohort H 6
Pre-eclampsia ANOVA 6
Table S5. ANOVA for preeclampsia. 6
References for supplement Information 7
Table S1. 8
Table S2. 9
Supplementary file descriptions 10
Supplementary Data 1. (Separate file) 10
Supplementary Data 2. (Separate file) 10
Supplementary Data 3. (Separate file) 10

Cohort overview

Samples used in this study were collected across 8 different academic centers. All studies have been approved by local IRBs, and informed consent was obtained from all study participants as part of the study protocols. This study only includes singleton pregnancies, we excluded individuals with systemic lupus erythematosus.

For Cohorts A and E, the preeclampsia cases were matched 2:1 on gestational age at blood draw, race, BMI and maternal age. To ensure that the preeclampsia classifier presented is representative of and applicable to a real-world setting, the non-case population was further expanded to include available samples from the gestational age analyses as well as samples with spontaneous preterm delivery. The control population thus includes normotensive, chronic hypertensive, gestational hypertensive and spontaneous preterm deliveries, this design was selected to make the control population reflective of a broader population.

Plasma samples were obtained from 8 different biobanks, we cannot guarantee material for all the same individuals are available for future replication efforts.

A detailed description of each cohort can be found below, and summary information can be found in main text table 1 and table S1

Labels used to describe race and ethnicity

In accordance with the latest guidelines on reporting race and ethnicity[46], we include a description of the composition of each of our labels, Asian, Black, Hispanic, White and Multiracial.

**Asian:**	Asian, Bangladeshi, Cambodian, Chinese, Far East Asian, Filipino, Indian, Japanese, Korean, Pacific Islander, Pakistani, South East Asian, Vietnamese
**Black:**	African, African American, AfroCaribbean, Black, Shirazi (Zanzibar Africans)
**Hispanic:**	Hispanic, Latino, Mexican, Non-Black Hispanic
**White:**	European, Middle Eastern, Spanish, White
**Multiracial:**	Asian and Black, Black and White, Cherokee and White, Filipino and Indian, Japanese and White, Mexican and Sioux and White, North/Central/South American Native and White, Panamanian and White, Taiwanese and White, Tlingit Tsimsian and White, rather not say, other, unclassified, unknown

Cohort descriptions

Cohort A[47]

LIFECODES is a prospective pregnancy biorespository that has been recruiting pregnant women in the greater Boston, MA area since 2006. Women 18 yrs. and older and plan to deliver at Brigham and Women's Hospital are eligible. Higher order pregnancies (triplets or greater) are excluded. To date N=5,569 pregnant women have been enrolled and followed, providing longitudinal samples and data, through delivery. Racial and ethnic makeup of LIFECODES follows the general US trend with 55% being Caucasian, 14.8% African American, 7.3% Asian, 18.4% Hispanic, and 4.5% Mixed/Other. The medical record for each subject in LIFECODES is independently reviewed by two certified Maternal Fetal Medicine physicians. Complications and outcomes for each subject are coded using a structured coding tool. The codes from each reviewer are then compared with disagreement in either pregnancy outcome or complication and is decided by a review committee.

Cohort B

The Global Alliance to Prevent Prematurity and Stillbirth (GAPPS) (www.gapps.org) has developed a continually recruiting cohort of pregnant women and their babies designed to combat the deficit of pregnancy-related specimens and accompanying data available for research.

Participants for this study were enrolled at all gestational ages from obstetric and antepartum clinic sites in Washington State under the Advarra IRB (FWA00023875) protocol number Pro00036408. Written informed consent was obtained from all participants and parental permission and assent were obtained for participating minors aged at least 15 years. A repository of biospecimens, including blood, collected longitudinally at each trimester of pregnancy and the postpartum period are linked to comprehensive patient data across the gestation. All blood is processed and stored at -80C within two hours of collection. The data repository was developed with the goal of supporting prematurity and stillbirth research and to better understand associated risk factors.

Comprehensive demographic, health history and dietary assessment surveys were administered, and relevant clinical data (for example, gestational age, height, weight, blood pressure, vaginal pH, diagnosis) were recorded. Relevant clinical information was obtained from neonates at birth and discharge and six weeks postpartum.

At subsequent prenatal visits, labor and delivery, and at discharge, characterizing surveys were administered, relevant clinical data were recorded and samples were collected. Vaginal and rectal samples were not collected at labor and delivery or at discharge. Women with any of the following conditions were excluded from sampling at a given visit: (1) Incapable of self-sampling due to mental, emotional or physical limitations; (2) More than minimal vaginal bleeding as judged by the clinician; (3) Ruptured membranes before 37 weeks; (4) Active herpes lesions in the vulvovaginal region; and (5) Experiencing active labor.

Key reference: https://www.gapps.org/Home/AboutBioservices

Cohort C[48]

Informed consent for sample and data collection was obtained at the University of Iowa by the Maternal Fetal Tissue Bank (IRB#200910784). Samples were prospectively collected from pregnant patients at the University of Iowa Hospitals & Clinics. Inclusion criteria included the ability to provide informed consent in English, pregnant, and being 18 years or older. Exclusion criteria are being HIV positive and/or Hepatitis C positive, being a prisoner, or being non-pregnant. Blood samples were collected in ACD-A tubes (Becton Dickinson). Plasma was aliquoted, snap frozen, and stored at -80C. All freezers are alarmed with temperature monitors. Time of sample collection and processing are recorded within the research information system managed by the UI Bioshare service (Labmatrix, Biofortis). All samples are coded and are annotated with clinical information.

Cohort D[49]

INSIGHT: Biomarkers to predict premature birth is an ongoing observational cohort study designed to study women at high risk of spontaneous preterm birth (sPTB) compared to low-risk controls. Plasma samples (taken between 16-23^{+6} weeks of gestation) provided for the current analyses were obtained from women with singleton pregnancies participants recruited from four tertiary antenatal clinics in the UK. High-risk pregnancies are defined by at least one of; prior sPTB or late miscarriage (between 16 to 37 weeks of gestation), previous destructive cervical surgery or incidental finding of a cervical length <25 mm on transvaginal ultrasound scan. Women with no risk factors for sPTB and otherwise well at the time of recruitment are recruited as low-risk controls from either routine antenatal or ultrasonography clinics at these centres. Exclusion criteria for both the high and low risk groups were multiple pregnancy, known major congenital fetal abnormality, rupture of membranes or current vaginal bleeding. Approval from London City and East Research Ethics Committee was granted (13/LO/1393). Informed written consent was obtained from all participants.

Cohort E[50]

The Pregnancy Outcomes and Community Health (POUCH) Study cohort includes 3,019 pregnant women enrolled at 16-27 weeks' gestation (1998-2004) from 52 clinics in five Michigan communities. Eligibility included singleton pregnancy and no known congenital anomaly, maternal age $\geq$ 15, maternal serum alpha-fetoprotein (MSAFP) screening, no pre-pregnancy diabetes mellitus, and English speaking. At enrollment study nurses interviewed participants and collected biologic samples (blood, urine, hair, vaginal fluid). An additional at-home data collection protocol included ambulatory blood pressure monitoring and three consecutive days of saliva and urine collection for measuring stress hormones. To conserve resources, a sub-cohort of 1,371 participants were studied in greater depth, i.e., medical records abstracted, biological samples analyzed, and placentas examined.[1] The sub-cohort is 42% primiparous, 57% 20-30 years of age, 42% African American and 49% non-Hispanic white, and 57% were insured through Medicaid.

Cohort F[51]

Samples were provided from biobanks collected in association with NIH P01 HD HD030367. These samples were part of 3 successive renewals of the PPG and collected between 2001 and 2012. In all cases samples were collected longitudinally across pregnancy from low risk pregnant women cared for at Magee-Womens Hospital Pittsburgh Pennsylvania. Exclusion criteria were pre-existing hypertension, diabetes, multiple gestation or renal disease. Charts were abstracted and reviewed by a jury of 5 clinicians. The population was approximately 50% African American, 50% Caucasian with very few other race/ethnicities included.

Cohort G[52]

The Pemba Pregnancy and Newborn Discovery Cohort (PPNDC) study is being undertaken in Pemba Island, Zanzibar, Tanzania. This ongoing study is follow-up continuation with methods similar to the AMANHI bio-repository study which involved 3 sites (Pakistan, Bangladesh and Pemba), methods already published[52].

The population is a mix of Arab and original Waswahili inhabitants of the island. A significant portion of the population also identifies as Shirazi people.

The main purpose of the study is to identify important biomarkers as predictors of important pregnancy–related outcomes and to expand the existing bio-bank in Pemba for future research as new methods and technologies become available.

Women of Reproductive Age (18-49 years), resident of the island who intended to stay in the study areas for the entire duration of follow–up and consented for collection of epidemiological data as well as biological samples are being enrolled in the study

Trained women fieldworkers (FWs), performed home visits every 2–3 months to all women of reproductive age in the study area to enquire about pregnancy. If a woman reported two or more consecutive missed period or suspected a pregnancy, FWs conducted a urine pregnancy test to confirm it. Pregnant women who provided consent underwent a screening ultrasound to date the pregnancy. All women in their early pregnancies with ultrasound confirmed gestational age between 8 and 19 weeks were consented for participation in the study. Women were randomized for antenatal maternal sample collection at either 24–28 weeks or 32–36 weeks gestation. The fathers of the babies also consented for their saliva sample collection.

A trained study worker conducted four home visits to all women in the cohort; at baseline (immediately after enrolment), at 24–28 weeks, 32–36 weeks and after 37 completed weeks of pregnancy to collect self-reported morbidity data from these women. Blood pressure and protein urea was measured by the study staff during these visits.

Bio-specimens, including blood, were collected from the pregnant women at the time of enrollment (between 8 and 19 weeks) and once during the antenatal period (24-28 or 32-26 weeks of gestation.

Cohort H[53]

This prospectively collected cohort from Roskilde hospital in Denmark, sampled participants 4 times during pregnancy at weeks 12, 20, 25 and 32. All Danish-speaking women over the age of 18 were eligible for inclusion. At each visit a blood sample was collected and we performed a detailed ultrasound examination. At end of collection in 2010 the cohort included a total of 1,214 participants.

Supplementary analyses

Gestational age ANOVA

ANOVA for gestational age was run to explore the impact of the model covariates BMI, maternal age and race/ethnicity.

Table S3. ANOVA for gestational age.

term	df	sumsq	meansq	statistic	p value	pctvarexp*
cfRNA	1	17005.8	17005.8	2778.4	5.67E-166	0.887
Race	3	76.63	25.54	4.17	0.0064	0.0040
BMI	1	1.64	1.64	0.27	0.61	8.54E-05
Age	1	0.037	0.037	0.006	0.94	1.95E-06
Residuals	341	2087.1	6.12	NA	NA	0.109

*pctvarexp, percent variance explained

Gestational age model without cohort correction

In this approach, we selected all samples from healthy pregnancies and split the dataset into a training set (80% of data) and a test set (20% of data), in which samples were stratified by cohort. Samples that did not pass QC filtering based on basic sequencing metrics had been previously excluded from analysis. We trained a Lasso model to predict the gestational age at collection for each sample using the mean absolute error as optimization metric and 10-fold cross-validation in the training set. We used all genes with mean log2(CPM+1) > 1 (12921 genes) plus a set of sequencing metrics as features for training. Modeling was performed in log2(CPM+1) space and all data was centered and scaled prior to modeling using the training set statistics. This led to a model with mean absolute error of 15.9 days in the with-hold test set using 487 transcriptomic features. We then selected the top 53 features of this model and retrained the Lasso using the same approach described above achieving a mean absolute error of 16.6 days in the held out test set.

Pairwise enriched gene sets in cohort H

We evaluated the strength of the fetal signal among significantly enriched gene sets for each of the pairwise comparisons between blood draws in cohort H. Significantly enriched gene sets were ranked in descending order based on abs(NES) * -log10(p-value) and over-representation of fetal gene sets was assessed based on a chance probability of observing a given number of fetal sets in 1 through *n* top ranked gene sets. The chance probability was determined using Fisher's exact test. The length of the longest list *n*, which was significantly enriched for fetal genes in every window between 1 and *n*, after correcting for the multiplicity of lists using Benjamini-Hochberg method, was determined for each pair of comparisons (Table S4).

Table S4. Differentially expressed gene sets in all pairwise comparisons between blood draws in cohort H

Blood draws compared	Longest significant list of ranked top affected gene sets (number of fetal sets)	Adjusted p-value	Total number of significantly enriched gene sets (number of fetal sets)
**2nd vs 1st**	81 (57)	0.037	197 (111)
**3rd vs 1st**	0	NA	266 (147)
**4th vs 1st**	0	NA	273 (145)
**3rd vs 2nd**	27 (20)	0.046	211 (109)
**4th vs 2nd**	0	NA	242 (124)
**4th vs 3rd**	0	NA	155 (87)

Pre-eclampsia ANOVA

ANOVA for preeclampsia was run to explore the impact of the model covariates BMI, maternal age and race/ethnicity.

Table S5. ANOVA for preeclampsia.

term	df	sumsq	meansq	F-statistic	p value	pctvarexp*
cfRNA	1	15.9	15.9	186	3.19E-36	0.267
Race	3	0.31	0.103	1.20	0.31	0.0052
Age	1	0.164	0.164	1.91	0.17	0.0028
BMI	1	0.19	0.19	2.19	0.14	0.0011
Residuals	503	43.2	0.086	NA	NA	0.724

*pctvarexp, percent variance explained

References for supplement Information

46. Flanagin, A., Frey, T., Christiansen, S. L., & AMA Manual of Style Committee. Updated Guidance on the Reporting of Race and Ethnicity in Medical and Science Journals. *JAMA* **326**, 621–627 (2021).
47. Lee, M. S. *et al.* Angiogenic markers in pregnancies conceived through in vitro fertilization. *Am J Obstet Gynecol* **213**, 212.e1–8 (2015).
48. Santillan, M. K. *et al.* Collection of a lifetime: a practical approach to developing a longitudinal collection of women's healthcare biological samples. *Eur J Obstet Gynecol Reprod Biol* **179**, 94–99 (2014).
49. Hezelgrave, N. L. *et al.* Cervicovaginal natural antimicrobial expression in pregnancy and association with spontaneous preterm birth. *Sci Rep* **10**, 12018 (2020).
50. Holzman, C., Senagore, P. K. & Wang, J. Mononuclear leukocyte infiltrate in extraplacental membranes and preterm delivery. *Am J Epidemiol* **177**, 1053–1064 (2013).
51. Powers, R. W. *et al.* Low placental growth factor across pregnancy identifies a subset of women with preterm preeclampsia: type 1 versus type 2 preeclampsia? *Hypertension* **60**, 239–246 (2012).
52. Baqui, A. H. *et al.* Understanding biological mechanisms underlying adverse birth outcomes in developing countries: protocol for a prospective cohort (AMANHI bio–banking) study. *Journal of Global Health* **7**, (2017).
53. Gybel-Brask, D., Høgdall, E., Johansen, J., Christensen, I. J. & Skibsted, L. Serum YKL-40 and uterine artery Doppler - a prospective cohort study, with focus on preeclampsia and small-for-gestational-age. *Acta Obstetricia et Gynecologica Scandinavica* **93**, 817–824 (2014).

Table S1.

Additional cohort information

Cohort label	Name	Location	Samples passing filters	Gestational Age at Blood Draw	Gestational Age at Delivery	Collection years*	Reference
A	LifeCodes	Boston, USA	201	24.0 +/-3.98	37.4 +/-3.15	2010-2019	[47]
B	GAPPS	Seattle, USA	385	26.3 +/-8.45	39.3 +/-1.08	2010-2018	www.gapps.org
C	Women's health tissue repository	Iowa City, USA	69	22.5 +/-5.04	39.3 +/-1.07	2010-2016	[48]
D	Insight	London, UK	186	20.0 +/-1.75	39.7 +/-1.22	2014-2019	[49]
E	POUCH	East Lansing, USA	353	21.8 +/-2.18	37.8 +/-3.83	1998-2004	[50]
F	PEPP	Pittsburgh, USA	793	22.8 +/-10.0	39.5 +/-1.10	1997-2013	[51]
G	PPNDC	Pemba, Tanzania	140	25.2 +/-9.66	39.8 +/-0.91	2014-2018	[52]
H	Roskilde	Roskilde, Denmark	412	22.5 +/-7.35	39.8 +/-1.19	2009-2010	[53]

*For samples included in this study, not for the original cohort which may extend beyond this range

Table S2.

Significantly enriched gene sets from the collection of cell type signature gene sets

Organ/ tissue/ fluid1	*Origin*	*Number of enriched gene sets*	*Absolute NES2*	*Adjusted3 p-value range*	*Set reference (PMID)*
Liver	*adult*	*28*	*1.95±0.09*	*8.2e-10 – 8.0e-3*	*31292543*
Developing heart	*fetal 5-25w*	*13*	*1.59±0.19*	*1.3e-09 –7.1e-3*	*31292543*
Olfactory	*adult*	*17*	*1.96±0.05*	*4.2e-06 – 8.4e-3*	*32066986*
Embryonic cortex	*fetal 22-23w*	*12*	*1.87±0.17*	*8.2e-10 – 5.0e-3*	*29867213*
Esophagus	*fetal 25w*	*2*	*1.71±0.19*	*3.8e-05 – 2.2e-3*	*29802404*
Large intestine	*fetal 24w*	*4*	*1.36±0.50*	*3.6e-06 – 4.2e-3*	*29802404*
Large intestine	*adult*	*3*	*2.01±0.40*	*8.2e-10 – 1.1e-7*	*29802404*
Small intestine	*fetal 24w*	*2*	*2.06±0.13*	*8.6e-08 – 2.3e-4*	*29802404*
Bone marrow	*adult*	*16*	*2.02±0.14*	*8.2e-10 – 9.8e-3*	*30243574*
Fetal retina	*fetal 5-25w*	*6*	*1.87±0.13*	*8.2e-10 – 5.6e-4*	*31269016*
Kidney	*adult*	*20*	*1.53±0.10*	*1.7e-09 – 7.2e-3*	*31249312*
Kidney	*fetal 12-19w*	*9*	*1.64±0.15*	*8.2e-10 – 7.9e-3*	*30166318*
Midbrain	*fetal and progenitor*	*21*	*1.62±0.09*	*8.2e-10 – 9.9e-3*	*27716510*
Pancreas	*adult*	*5*	*1.47±0.29*	*8.2e-10 – 1.2e-4*	*27693023*
Cord blood	*adult and progenitor*	*5*	*2.02±0.21*	*8.2e-10 – 1.4e-3*	*29545397*
Prefrontal cortex	*fetal 8-26 w*	*13*	*2.02±0.12*	*8.2e-10 – 7.7e-3*	*29539641*
Eye epithelial	*fetal (Descartes)*	*3*	*1.94±0.06*	*8.2e-10 – 6.7e-8*	*33184181*
Heart endocardial	*fetal (Descartes)*	*2*	*1.80±0.42*	*5.2e-6 – 4.2e-2*	*33184181*
Liver erythroblast	*fetal (Descartes)*	*1*	*1.51*	*3.2e-3*	*33184181*
Lung	*fetal (Descartes)*	*2*	*1.73±0.14*	*1.1e-9 – 8.3e-7*	*33184181*
Pancreas myeloid	*fetal (Descartes)*	*1*	*1.53*	*2.8e-3*	*33184181*
Placenta	*fetal (Descartes)*	*4*	*1.98±0.12*	*8.2e-08 – 1.9e-4*	*33184181*
Skeletal muscle	*fetal (Descartes)*	*2*	*2.01±0.12*	*8.1e-08 – 1.9e-4*	*33184181*
Stomach squamous epithelial	*fetal (Descartes)*	*1*	*1.85*	*4.3e-5*	*33184181*
Vascular endothelial	*fetal (Descartes)*	*4*	*1.80±0.14*	*4.22e-6 – 5.12e-4*	*33184181*

[1] - Listed in the order of sets in C8 collection (v7.2) at http://www.gsea-msigdb.org/ followed by Descartes gene sets in alphabetical order, [2] NES - normalized enrichment score; [3] - *p*-values were adjusted using Benjamini-Hochberg procedure

Supplementary file descriptions

Supplementary Data 1. (Separate file)

Full list of features selected by the lasso for the gestational age model, including their weights in the model.

Supplementary Data 2. (Separate file)

Full list of gene sets with significant negative correlation to gestational age in at least 3 independent cohorts. Gene sets were discovered in cohort H and confirmed in at least 2 of cohorts A, B or G. Slope is average change in gene set expression in CPM/week. Adjusted p-value for discovery cohort. Number of cohorts with significant signal, including discovery cohort. Type, origin of data set, fetal or adult tissue. Additional sheets for component genes for each set of adult or fetal genes.

Supplementary Data 3. (Separate file)

Full list of gene sets with significant positive correlation to gestational age in at least 3 independent cohorts. Gene sets were discovered in cohort H and confirmed in at least 2 of cohorts A, B or G. Slope is average change in gene set expression in CPM/week. Adjusted p-value for discovery cohort. Number of cohorts with significant signal, including discovery cohort. Type, origin of data set, fetal or adult tissue. Additional sheets for component genes for each set of adult or fetal genes.
