# Supplementary material for: RNA profiles reveal signatures of future health and disease in pregnancy
Source: Nature. 2022 Jan 5;601(7893):422–7. doi: 10.1038/s41586-021-04249-w (PMC8770117; doi:10.1038/s41586-021-04249-w)
Supplement: Supplementary file 2 — Reporting Summary [file 41586_2021_4249_MOESM2_ESM.pdf]

nature portfolio

Corresponding author(s): Morten Rasmussen, Michal A Elovitz, Thomas F McElrath

Last updated by author(s): Oct 27, 2021

Reporting Summary

Nature Portfolio wishes to improve the reproducibility of the work that we publish. This form provides structure for consistency and transparency in reporting. For further information on Nature Portfolio policies, see our Editorial Policies and the Editorial Policy Checklist.

Statistics

For all statistical analyses, confirm that the following items are present in the figure legend, table legend, main text, or Methods section.

n/a	Confirmed	
☐	☒	The exact sample size (*n*) for each experimental group/condition, given as a discrete number and unit of measurement
☐	☒	A statement on whether measurements were taken from distinct samples or whether the same sample was measured repeatedly
☐	☒	The statistical test(s) used AND whether they are one- or two-sided<br>*Only common tests should be described solely by name; describe more complex techniques in the Methods section.*
☐	☒	A description of all covariates tested
☐	☒	A description of any assumptions or corrections, such as tests of normality and adjustment for multiple comparisons
☐	☒	A full description of the statistical parameters including central tendency (e.g. means) or other basic estimates (e.g. regression coefficient) AND variation (e.g. standard deviation) or associated estimates of uncertainty (e.g. confidence intervals)
☐	☒	For null hypothesis testing, the test statistic (e.g. *F*, *t*, *r*) with confidence intervals, effect sizes, degrees of freedom and *P* value noted<br>*Give P values as exact values whenever suitable.*
☒	☐	For Bayesian analysis, information on the choice of priors and Markov chain Monte Carlo settings
☒	☐	For hierarchical and complex designs, identification of the appropriate level for tests and full reporting of outcomes
☒	☐	Estimates of effect sizes (e.g. Cohen's *d*, Pearson's *r*), indicating how they were calculated

Our web collection on statistics for biologists contains articles on many of the points above.

Software and code

Policy information about availability of computer code

Data collection	N/A
Data analysis	trimmomatic 0.36-6; star 2.6.1a_08-27; picard.jar 2.18.3-SNAPSHOT; htseq-count 0.11.2; R 4.0.3; R 4.0.4; python 3.7; python 3.9.1; jupyter 6.2; tidyverse 1.3.0; Rstudio 1.4.1106; bioconductor 3.12; fgsea 1.16.0; DESeq2 1.30.1<br>Reference genome: GRCh38_ensembl

For manuscripts utilizing custom algorithms or software that are central to the research but not yet described in published literature, software must be made available to editors and reviewers. We strongly encourage code deposition in a community repository (e.g. GitHub). See the Nature Portfolio guidelines for submitting code & software for further information.

Data

Policy information about availability of data

All manuscripts must include a data availability statement. This statement should provide the following information, where applicable:

- Accession codes, unique identifiers, or web links for publicly available datasets
- A description of any restrictions on data availability
- For clinical datasets or third party data, please ensure that the statement adheres to our policy

The data and code that support the findings of this study are available from the corresponding author upon reasonable request under reasonable terms with permission from relevant third parties, however some of the data and code may not be publicly available, including due to restrictions pertaining to participant privacy and consent and information and obligations to third parties.

Field-specific reporting

Please select the one below that is the best fit for your research. If you are not sure, read the appropriate sections before making your selection.

☒ Life sciences ☐ Behavioural & social sciences ☐ Ecological, evolutionary & environmental sciences

For a reference copy of the document with all sections, see nature.com/documents/nr-reporting-summary-flat.pdf

Life sciences study design

All studies must disclose on these points even when the disclosure is negative.

Sample size	No sample size calculation was performed.
Data exclusions	qPCR of ACTB as well as MultiQC sequencing metrics were monitored to eliminate sample outliers before performing gene expression analyses. Individual samples more than 3 standard deviations from the mean were removed as outliers. A total of 193 of 2,732 samples (7.1%) were removed following this filtering.
Replication	Each sample is a single aliquot of human plasma and volume only allows for one extraction, so sample reproducibility cannot be confirmed
Randomization	For gestational age analyses samples were split into 80% training and 20% test sets. These were stratified by gestational age to ensure even distribution in both training and held-out test set.
Blinding	Sample labels were not blinded to analyses team. In a leave-one-out cross-validation blinding is not possible.

Reporting for specific materials, systems and methods

We require information from authors about some types of materials, experimental systems and methods used in many studies. Here, indicate whether each material, system or method listed is relevant to your study. If you are not sure if a list item applies to your research, read the appropriate section before selecting a response.

Materials & experimental systems

n/a	Involved in the study	
☒	☐	Antibodies
☒	☐	Eukaryotic cell lines
☒	☐	Palaeontology and archaeology
☒	☐	Animals and other organisms
☐	☒	Human research participants
☒	☐	Clinical data
☒	☐	Dual use research of concern

Methods

n/a	Involved in the study	
☒	☐	ChIP-seq
☒	☐	Flow cytometry
☒	☐	MRI-based neuroimaging

Human research participants

Policy information about studies involving human research participants

Population characteristics	We intentionally targeted a diverse racial and ethnic composition of our samples and globally have 3.8% Asian, 32.6% Black, 5.4% Hispanic, 55.1% White and 3.1% mixed/unknown/not reported. For most samples we have data on maternal age, pre-pregnancy BMI, and preeclampsia status.
Recruitment	This is a retrospective study of prospectively collected samples from 8 different cohorts. We selected cohorts based on literature search of pregnancy cohorts with EDTA plasma stored at -80C. Recruitment criteria for individual cohorts are reported in the literature.
Ethics oversight	All cohorts have previously been published on, references to relevant IRB approvals for individual cohorts available through references in supplementary text.

Note that full information on the approval of the study protocol must also be provided in the manuscript.
