# Supplementary material for: RNA profiles reveal signatures of future health and disease in pregnancy
Source: Nature. 2022 Jan 5;601(7893):422–7. doi: 10.1038/s41586-021-04249-w (PMC8770117; doi:10.1038/s41586-021-04249-w)
Supplement: Supplementary file 3 — Peer Review Information [file 41586_2021_4249_MOESM3_ESM.pdf]

Peer Review File

Manuscript Title: RNA profiles reveal signatures of pregnancy's future health and disease

Reviewer Comments & Author Rebuttals

Reviewer Reports on the Initial Version:

Referee #1 (Remarks to the Author):

In this paper, Rasmussen and colleagues demonstrate that cfRNA signatures in maternal blood from a large, racially- and ethnically-diverse cohort can accurately date the pregnancy with comparable accuracy to ultrasound, can help monitor the development of specific organs, namely small intestine, kidney and heart, and may offer signals with respect to early (2nd trimester) detection of preeclampsia. This manuscript builds upon and validates findings described in a 2018 publication (Ngo et al, Science, 2018) that found in a pilot of 31 pregnant individuals that cfRNA transcripts in maternal blood predicted gestational age with comparable accuracy to ultrasound, and in 38 pregnant individuals, seven cfRNA transcripts in maternal blood could predict preterm birth up to 2 months prior to preterm delivery. This manuscript also builds upon findings described in a s2014 manuscript demonstrating the potential of cfRNA in maternal circulation to monitor fetal-specific tissue transcripts (largely originating from placenta; Koh et al, PNAS, 2014).

Validity: no concerns, robust study design with large validation cohort

Originality: The large cohort size and substantial racial and ethnic diversity are strengths of the study, but the concepts of determining gestational age via cfRNA in maternal blood, monitoring fetal development via cfRNA in maternal blood, and early biomarkers for later-onset preeclampsia are at this point not novel in and of themselves (reported in prior publications as cited above). Numerous other studies have investigated the test characteristics of early biomarkers/predictive markers for later development of preeclampsia, including gene expression signatures and other omics approaches (there are numerous, just a few examples: Banadakoppa et al, Sci Reports, 2020; Tarca et al, J Maternal-Fetal Neonat Med, 2019; Odibo et al, Prenat Diagn, 2011; one even by one of the same authors as this paper- Yadama et al, Sci Reports, 2020). The positive predictive value of 32.3% for preeclampsia is not incredibly high (FFN for predicting preterm birth has a PPV ranging from 17-30% depending on studies, by comparison), and likely the PPV would be slightly lower in other cohorts with a more typical occurrence of preeclampsia (e.g. 10% versus 13%). Thus, it's not clear that this represents any sea-change in the monitoring for or care of pregnancies at risk for preeclampsia.

Data and Methodology:
1) Page 5, lines 183-207: The term "fetal organ signal" is imprecise and doesn't address some key questions a reader is left with after the manuscript: (1) if these transcripts truly reflect fetal organ development and not just the placenta, then how might these fetal organ-specific transcripts be getting into maternal blood, and (2) how can the authors be sure these transcripts aren't just reflecting apoptosis of a specialized set of placental cells, rather than true fetal organ development. CfRNA in maternal blood has been demonstrated to largely originate from the placenta/placental apoptosis, even in a manuscript by some of the same authors in 2014. Placental-origin transcripts also would be expected to increase progressively across gestation, so that aspect is not particularly convincing that these transcripts are shedding directly off fetal organs. In addition, maternal cardiac and renal transcripts also could certainly be changing across gestation, so more detail is needed on how these transcripts are known to reflect the development of fetal organs rather than maternal or placental. Some more discussion of the origin and trafficking of cf fetal RNA (cffRNA) in maternal circulation is warranted.
2) Do any of the participants included here have overlap with participants included in other publications by some members of the same group (e.g. the Science 2018 paper)? Or are these all

new participants?
3) Page 5, lines 183-190: Are all of the organ-specific transcript results based on 95 women? While the text mentions N=95 women who had data available for all 4 collections, the figure mentions N=91, this should be reconciled. Were the additional 3 cohorts A, B, G also used to try to discover more organ-specific transcripts? Or only to verify increase of the organ-specific transcripts identified in the original 95 across gestation?
4) It's interesting if fetal organ transcripts are detectable in maternal circulation that there would be no gestational-age-specific or evolving pulmonary or brain signal across gestation, as those organs are known to continue to mature significantly across gestation. Why do the authors think there was no signal from other organs that would be of significant interest with respect to non-invasive monitoring of their maturation/function?
5) Functional analyses of the differentially-expressed genes across gestation that are reportedly reflecting small intestine, renal and cardiac development would enrich the manuscript and provide potentially important insights/increase significance.
6) Page 5, line 218 on- discussion of predictive test for preeclampsia in 2nd trimester blood: Several points merit clarification/additional information
a) Knowledge of when the participants developed preeclampsia relative to when the 2nd trimester blood sample was taken is very relevant to gauging how much of an advance this test could be, and how it differs substantively from other 3rd trimester tests which detect preeclampsia very close to when clinical features would declare themselves. Women can certainly develop preeclampsia at 27 weeks and close to 27 weeks, so a supplemental figure depicting when each of the preeclampsia participants had blood drawn versus when they developed preeclampsia would be very useful, as well as median (IQR) for time from blood draw to development of preeclampsia (this could be in Table 1). It would also be helpful if Table 1 showed how many of the preeclampsia cases and controls came from which cohort.
b) The use of an old definition of preeclampsia (based on 2013 rather than updated 2017 definitions) is potentially problematic. Why was this older definition used? If participants were properly classified using up to date preeclampsia definition, would the results hold? Would any of the "gestational hypertension" control cases have met the 2017 definition for preeclampsia by lab abnormalities?
c) Given that at least 3 of the 7 preeclampsia RNA markers are related to embryo implantation, early miscarriage, and early placental development (CLDN7, TLE6, PAPPA2), it seems to make sense to see how the panel of 7 performs for predicting preeclampsia from 1st trimester blood draws. Figure 1 suggests there are plenty of first trimester samples, so there must be a reasonable number of preeclampsia cases who have first trimester samples to test. Is there a reason this evaluation of marker robustness for an earlier timepoint in pregnancy (potentially at an intervenable period with baby aspirin initiation) was not performed?
d) What were the 452 non-cases of preeclampsia "matched" to the preeclampsia cases on the basis of? 452 is not a multiplier of 72, so unclear how the matching was performed and details are not provided.
e) Page 7, lines 267-270: "both aligned well with what is known about preeclampsia pathophysiology" is too general of a statement. How does downregulated gene sets related to immune pathways align with what is known about preeclampsia specifically, for example?
f) The discussion of the 7 genes that predict preeclampsia should include how this set of 7 genes overlaps or doesn't overlap with lists generated by Munchel et al (7) or Moufarraj et al (9).

Appropriate use of statistics and treatment of uncertainties: No concerns

Conclusions: As referenced above, the utility and novelty of this second trimester test for preeclampsia is somewhat difficult to gauge, given the relatively low PPV, use of outdated definitions of preeclampsia, and lack of information about how long the blood was drawn before clinical symptoms manifested. Given the low PPV, the statement "a cfRNA platform enables early detection of multiple clinically relevant endpoints (e.g. gestational age and preeclampsia) from a single specimen…" (lines 327-28) is overstated. The strongest evidence offered in the paper is a confirmation/validation of the 2018 paper in Science demonstrating relatively robust prediction of

gestational age +/- 2 weeks from cfRNA in a maternal blood sample. The evidence that cfRNA can be used to predict preeclampsia in a meaningful way and to monitor fetal organ development is less robust, for the reasons detailed above.
In this paper, Rasmussen and colleagues demonstrate that cfRNA signatures in maternal blood from a large, racially- and ethnically-diverse cohort can accurately date the pregnancy with comparable accuracy to ultrasound, can help monitor the development of specific organs, namely small intestine, kidney and heart, and may offer signals with respect to early (2nd trimester) detection of preeclampsia. This manuscript builds upon and validates findings described in a 2018 publication (Ngo et al, Science, 2018) that found in a pilot of 31 pregnant individuals that cfRNA transcripts in maternal blood predicted gestational age with comparable accuracy to ultrasound, and in 38 pregnant individuals, seven cfRNA transcripts in maternal blood could predict preterm birth up to 2 months prior to preterm delivery. This manuscript also builds upon findings described in a s2014 manuscript demonstrating the potential of cfRNA in maternal circulation to monitor fetal-specific tissue transcripts (largely originating from placenta; Koh et al, PNAS, 2014).

Validity: no concerns, robust study design with large validation cohort

Originality: The large cohort size and substantial racial and ethnic diversity are strengths of the study, but the concepts of determining gestational age via cfRNA in maternal blood, monitoring fetal development via cfRNA in maternal blood, and early biomarkers for later-onset preeclampsia are at this point not novel in and of themselves (reported in prior publications as cited above). Numerous other studies have investigated the test characteristics of early biomarkers/predictive markers for later development of preeclampsia. The positive predictive value of 32.3% is not incredibly high (FFN for predicting preterm birth has a PPV ranging from 17-30% depending on studies, by comparison), and likely would even be slightly lower in other cohorts with a more typical occurrence of preeclampsia (e.g. 10% versus 13%). Thus, it's not clear that this represents any sea-change in the monitoring for or care of pregnancies at risk for preeclampsia.

Data and Methodology:
1) Page 5, lines 183-207: The term "fetal organ signal" is imprecise and doesn't address some key questions a reader is left with after the manuscript: (1) if these transcripts truly reflect fetal organ development and not just the placenta, then how might these fetal organ-specific transcripts be getting into maternal blood, and (2) how can the authors be sure these transcripts aren't just reflecting apoptosis of a specialized set of placental cells, rather than true fetal organ development. CfRNA in maternal blood has been demonstrated to largely originate from the placenta/placental apoptosis, even in a manuscript by some of the same authors in 2014. Placental-origin transcripts also would be expected to increase progressively across gestation, so that aspect is not particularly convincing that these transcripts are shedding directly off fetal organs. In addition, maternal cardiac and renal transcripts also could certainly be changing across gestation, so more detail is needed on how these transcripts are known to reflect the development of fetal organs rather than maternal or placental. Some more discussion of the origin and trafficking of cf fetal RNA (cffRNA) in maternal circulation is warranted.
2) Do any of the participants included here have overlap with participants included in other publications by some members of the same group (e.g. the Science 2018 paper)? Or are these all new participants?
3) Page 5, lines 183-190: Are all of the organ-specific transcript results based on 95 women? While the text mentions N=95 women who had data available for all 4 collections, the figure mentions N=91, this should be reconciled. Were the additional 3 cohorts A, B, G also used to try to discover more organ-specific transcripts? Or only to verify increase of the organ-specific transcripts identified in the original 95 across gestation?
4) It's interesting if fetal organ transcripts are detectable in maternal circulation that there would be no gestational-age-specific or evolving pulmonary or brain signal across gestation, as those organs are known to continue to mature significantly across gestation. Why do the authors think there was no signal from other organs that would be of significant interest with respect to non-invasive monitoring of their maturation/function?

5) Functional analyses of the differentially-expressed genes across gestation that are reportedly reflecting small intestine, renal and cardiac development would enrich the manuscript and provide potentially important insights/increase significance.
6) Page 5, line 218 on- discussion of predictive test for preeclampsia in 2nd trimester blood: Several points merit clarification/additional information
a) Knowledge of when the participants developed preeclampsia relative to when the 2nd trimester blood sample was taken is very relevant to gauging how much of an advance this test could be, and how it differs substantively from other 3rd trimester tests which detect preeclampsia very close to when clinical features would declare themselves. Women can certainly develop preeclampsia at 27 weeks and close to 27 weeks, so a supplemental figure depicting when each of the preeclampsia participants had blood drawn versus when they developed preeclampsia would be very useful, as well as median (IQR) for time from blood draw to development of preeclampsia (this could be in Table 1). It would also be helpful if Table 1 showed how many of the preeclampsia cases and controls came from which cohort.
b) The use of an old definition of preeclampsia (based on 2013 rather than updated 2017 definitions) is potentially problematic. Why was this older definition used? If participants were properly classified using up to date preeclampsia definition, would the results hold? Would any of the "gestational hypertension" control cases have met the 2017 definition for preeclampsia by lab abnormalities?
c) Given that at least 3 of the 7 preeclampsia RNA markers are related to embryo implantation, early miscarriage, and early placental development (CLDN7, TLE6, PAPPA2), it seems to make sense to see how the panel of 7 performs for predicting preeclampsia from 1st trimester blood draws. Figure 1 suggests there are plenty of first trimester samples, so there must be a reasonable number of preeclampsia cases who have first trimester samples to test. Is there a reason this evaluation of marker robustness for an earlier timepoint in pregnancy (potentially at an intervenable period with baby aspirin initiation) was not performed?
d) What were the 452 non-cases of preeclampsia "matched" to the preeclampsia cases on the basis of? 452 is not a multiplier of 72, so unclear how the matching was performed and details are not provided.
e) Page 7, lines 267-270: "both aligned well with what is known about preeclampsia pathophysiology" is too general of a statement. How does downregulated gene sets related to immune pathways align with what is known about preeclampsia specifically, for example?
f) The discussion of the 7 genes that predict preeclampsia should include how this set of 7 genes overlaps or doesn't overlap with lists generated by Munchel et al (7) or Moufarraj et al (9).

Appropriate use of statistics and treatment of uncertainties: No concerns

Conclusions: As referenced above, the utility and novelty of this second trimester test for preeclampsia is somewhat difficult to gauge, given the relatively low PPV, use of outdated definitions of preeclampsia, and lack of information about how long the blood was drawn before clinical symptoms manifested. Given the low PPV, the statement "a cfRNA platform enables early detection of multiple clinically relevant endpoints (e.g. gestational age and preeclampsia) from a single specimen…" (lines 327-28) is overstated. The strongest evidence offered in the paper is a confirmation/validation of the 2018 paper in Science demonstrating relatively robust prediction of gestational age +/- 2 weeks from cfRNA in a maternal blood sample. The evidence that cfRNA can be used to predict preeclampsia in a meaningful way and to monitor fetal organ development is less robust, for the reasons detailed above.

Suggested improvements: would be performed in response to concerns raised above.

References: no concerns

Clarity and context: clearly written, appropriate abstract, some of conclusions read as overstated as previously described.

Referee #2 (Remarks to the Author):

The goal of this study was to demonstrate the utility of cell free signatures in 3 contexts: (1) gestational age estimation (2) monitoring fetal organ development and (3) as a biomarker for preeclampsia. Data was collected across eight different cohorts in over 1840 participants, representing a very large sample size. Overall, the researchers are using a very novel method to tackle a number of challenging problems in pregnancy. The blood test for gestational length has broad applicability, particularly in low resource settings. Each concept alone would be very valuable, but by grouping all three analyses together, a lot of key details are missing, and analyses are presented in a very shallow way. The supplemental methods section is really hard to follow, as it doesn't follow a logical progression, and is missing a lot of details. Some parts of the statistical analyses seem valid (such as the cell free RNA data generation, and the gene set enrichment analyses), but without more detail about the underlying data, it is hard to understand how much these findings are impacted by batch effects. Moreover, one of the biggest strengths of this paper is the strong ethnic and racial diversity of the cohort, but this data isn't analyzed or presented appropriately.

Part 1: Cohorts Involved
1. The authors provide limited information on the cohorts in table 1, with more detailed information provided in the supplemental materials. I find the presentation of cohorts A-H very confusing, and would prefer if the co-authors just referred to the individual cohorts by name or acronym, or at least presented this information in part in supplemental table 1.

2. This manuscript is using a number of different cohorts that are from very different parts of the world, and with data collected at very different timepoints, and with very different goals. It would be helpful to clarify this in the manuscript text, and in table one to clarify the dates of cohort collection and/or if collection is still ongoing. This is relevant because some of these studies span from 1998 to the present day, and there are a number of fundamental changes that have occurred in prenatal care and the treatment of gestational diseases within this timespan.

3. The authors present the exclusion/inclusion criteria for each study individually. However, was there a unifying set of inclusion/exclusion criteria beyond each study that was used for the paper itself. Or any sub-analyses? For example, for measurements of gestational length, I would exclude all multi-fetal pregnancies due to inherent differences in gestational length in these pregnancies. Overall, the clarity of this manuscript could be improved by adding a supplemental figure/table to the paper that follows the STROBE guidelines through a STROBE diagram.

4. One of the largest strengths that the authors highlight is the diversity of the cohorts, however this is not true for all datasets. For example-Dataset H was collected in Denmark and is almost entirely white, with no other races presented. This is significant because The molecular signatures for fetal organ development only uses data from cohort H (Line 187).

5. The authors group race and ethnicity together in their descriptions and analyses in Table 1. Race and Ethnicity are 2 separate constructs and should be presented independently. The authors could also stratify into something like-black Hispanic, white Hispanic, white-non Hispanic, black non Hispanic as an alternative.

Part 2: General RNA sequencing Comments
6. Since the authors are combining data from so many cohorts and timepoints, there are bound to be substantial batch effects. The authors mention in their study design that each cohort was treated as an independent batch and a batch correction was applied prior to modeling the data. (Line 117) However, there is no mention off this in the supplemental data at all. The authors discuss feature normalization, but never present any effects of batch on results. There is one figure (line 686) that has batch effects for just one gene, but there should be some sort of PCA

plot showing batch effects across all genes, as they have done to show a lack of variation associated with the data to investigate how covariates influenced gestational length and preeclampsia . Also, in this plot they mention that each cohort has its own color but the cohorts aren't labeled and the choice of color is not optimal for categorical data. I am also wondering if the authors performed separate batch normalizations before or after processing their data.

7. Along the lines of batch effects, it seems like there are layers of batch effects present here. For example, the GAPPS biorepository collects from 2 different locations, introducing batch effects at the cohort level. Moreover, with this many samples could not have been analyzed in one RNA sequencing run, so there also must be batch effects within the sequencing runs.

Part 2: Molecular signature of Gestational Age
8. The wording of this "gestational age" is very confusing. Are the authors predicting gestational age at the timepoint of sample collection or at the timepoint of delivery. In the abstract and at some parts in the manuscript, they simply refer to gestational age, but at other points they refer to gestational age progression (line 96). Adding a sentence and tightening up the language could clarify this

9 .The authors strategies of performing cross validation in the training dataset and then testing in the test dataset for lasso regression are valid. Can the authors show the distribution of sample information (as in table 1) for the training and testing set? What cohorts were the samples taken from?

10. It is hard to understand how the clinical factors could impact the model construction without knowing what cohorts the model was constructed from. The authors state that inclusion of clinical factors did not improve the model much, which is surprising. The authors only include maternal BMI, maternal age, and maternal race/ethnicity. However, these list of covariates (for this analysis and others) are not inclusive of all factors that might impact gestational length. I am surprised the authors don't have a metric for maternal socio-economic status, such as maternal education or maternal income. I understand it would be challenging to come up with a harmonized variable across cohorts, but without a marker of SES it hard to interpret.

11. In the table presented (line 707), what does pctvarexp stand for? I am presuming it is percent variation explained, but this should be spelled out (as well as for all presented on here).

12. It it would be useful to present in a supplemental table what the features that best predicted gestational length are, or (if this is proprietary) the authors could provide some insight into the biological functions.

Molecular Signatures of Fetal Organ Development

13. Supplemental Figure S1 does a nice job of showing trends in key pregnancy endocrine pathways across pregnancy. However, the Y axis does not make sense because they are all the same number, likely due to a plotting issue.

14. What exactly do the authors mean when they say "analyzing gene sets and by targeting gene sets" (line 185). How were these gene sets defined? It seems odd to me to use GO pathways to do this, when there are databases of tissue specific genes. However, in supplemental Table S1 they list a number of papers. How were these papers selected?

15. How are the authors defining significant changes in gene sets (up or down) (Lines 214-216).

16. Supplemental Figure S1-Again the axes here do not really make sense. Additionally, if the authors were to restrict to a smaller window of space on the Y axis, it would be easier to see changes.

Classifying risk of preeclampsia

18. What does NES stand for in supplemental figure S4. Also, please check the spelling of the titles. Moreover, the authors only present the top 20 genes, but since they are mentioning 88 significant fetal gene sets (Line 738), it would be helpful to present the full results in a supplemental table.

19. Line 226: what was the criteria for matching? Were there a matched % of preterm birth samples? How was the data distributed throughout the cohort? A table here showing this data would be helpful.

20. The authors perform an ANOVA on a very limited number of covariates and claim that they do not influence risk of preeclampsia. This is pretty surprising, given the breadth of studies showing that Black women at are at higher risk of preeclampsia. A more in depth analysis would be required, including reporting the number of cell free RNA transcripts associated with maternal race and ethnicity alone, and a description of how batch effects were handled. The authors should also consider adjusting for other confounders associated with increased risk of preeclampsia, such as a history of preeclampsia, gravidity.

21. How does this model of preeclampsia compare to other models of preeclampsia using omics data?

Discussion:
22. It remains unclear if an ANOVA and correlation of the first 2 PCs alone is enough to conclude that there is not confounding by race/ethnicity, as the authors claim (Line 296). I think this sort of conclusion could only be made if the models were run with and without these covariates.

23. It seems odd to me to group these 3 separate analyses in one paper, and not compare and contrast or build the cell-free RNA signatures against each other into a cohesive story.

24. This manuscript is hard to follow, but more specific titles, both in the figures and in the headings of the supplemental text, would make things more clear

Referee #3 (Remarks to the Author):

Rasmussen et al have expanded on their earlier work to further explore the potential clinical value of cfRNA analysis in maternal blood. Three pregnancy-related facets have been investigated in this study:
1. the feasibility to estimate the gestational age by cfRNA analysis
2. Whether cfDNA profile would offer information regarding fetal development
3. cfRNA analysis as predictive markers of preeclampsia

These investigations were performed on samples dervied from 8 recruitment cohorts. Because all the analyses have been performed retrospectively in batch formats and no prospective interventions had been applied to any of the cohorts, describing the samples as having been prospectively collected in the manner shown in lines 107 to 109 is somewhat misleading.

Regarding the facet on gestational age prediction, the authors have been able to improve on their previous work published as Ngo et al Science 2018. In Ngo et al, gestational age estimation for each pregnancy was performed using the levels of certain cfRNA transcripts taken across several dates. In the present work, the authors achieved gestational age estimation at errors within ~ 15

days with the use of just one blood sample. By performing the feature mining on a racially and ethnically diverse sample set, the authors showed that the resultant metric was mostly dependent on gestational age. Other demographic factors contributed to < 1% variance.

The conclusions drawn for this part of the study are robust which is contributed by having a subset of IVF pregnancies to verify the accuracy of the gestational age prediction; a relatively large sample size; segregating the sample cohort into training and validation sets; as well as checking for evidence to exclude the possibility of over-fitting.

However, one interpretation of the data has been overstated. In lines 176 to 177, the authors seem to be forecasting the utility of the gestational age prediction algorithm to contribute to the clinical management of small-for-gestational-age fetuses. This is too much of a leap of faith because the dataset did not seem to have included such pregnancies. It is not known if the prediction algorithm would work equally well in such pregnancies whereby the suboptimal fetal growth might be due to underlying pathologies that could alter the cfRNA profiles.

Regarding the second facet of the study, gene sets considered by gene ontology to be associated with fetal small intestine and developing heart were observed to increase with gestational age advancement while the gene set identified as associated with nephron progenitor was observed to reduce with progressive gestational age. Based on these gestational-age related trends, the authors interpreted the data as providing a window into fetal development (line 216). It is somewhat premature to make this conclusion when the associations of the sets of cfRNA transcripts with fetal intestinal / heart / kidney development have not been verfied using samples with fetuses showing developmental issues in those organs. Some of the authors have also reported the detection of "fetal brain" transcripts in maternal plasma in Koh et al Proc Natl Acad Sci U S A 2014.

Furthermore, the data mining was performed on timed serial samples collected from 95 women as part of cohort H which according to Figure 1 is a homogenous population of one ethnic/racial group. This is a much smaller and much less diverse sample set than what the authors have described their study design as more generalizable due to the sample size and diverse populations (lines 106-107).

The third part of the study involved 72 cases of preeclampsia and 452 controls from cohorts A and E. Using a panel of cfRNA transcripts, a sensitivity of 75% and a PPV of 32.3% were achieved. It would be useful to know and stratify the preeclampsia cases into the commonly used clinical phenotypes, namely late vs early onset, severity and if asprin has been administered early in gestation. With or without the stratification, having 72 cases of preeclampsia still falls within the tens of cases the authors considered as limitations of the prior studies (line 300).

In fact, in the first two paragraphs of the discussion, the key advance of the present study was identified as the large sample size and broad diversity of the study cohorts. Yet, this advantage mainly rests with the first facet of the study and less so for the other two parts.

In summary, cfRNA analysis may offer much potential for monitoring pregnancy health. The present study is definitely a step in the right direction. The manuscript is succinctly written with the data and methods clearly presented. However, there are quite a number of overstatements, priority or unqualified statements. For example, the advantage of the size and composition of the sample cohorts have been too broadly generalized to all facets of the study. Another example of a unqualified statement is that claim in lines 83 to 85 indicating an early cfRNA test would contribute to advancements in fetal maternal health globally. Nothing in the present manuscript is clinic-ready. The manuscript title is also unjustified suggesting the work offers a molecular window into maternal fetal health. The only part that may be relevant to "maternal health" is perhaps the mentioning of preeclampsia might put the mother at lifetime risk of cadiovascular consequences. No data in the manuscript showed that one could prognosticate the mother or rule out maternal

disease. The authors seemed to suggest what has been observed in the use of liquid biopsy for breast cancer could predict the clinical value of pregnancy cfRNA tests (lines 338 to 340). As for "fetal health", there is not enough evidence in the manuscript to show clinically actionable differences to ensure or maintain fetal health using the cfRNA data.

Author Rebuttal to Initial Comments:

Referee #1 (Remarks to the Author):

In this paper, Rasmussen and colleagues demonstrate that cfRNA signatures in maternal blood from a large, racially- and ethnically-diverse cohort can accurately date the pregnancy with comparable accuracy to ultrasound, can help monitor the development of specific organs, namely small intestine, kidney and heart, and may offer signals with respect to early (2nd trimester) detection of preeclampsia. This manuscript builds upon and validates findings described in a 2018 publication (Ngo et al, Science, 2018) that found in a pilot of 31 pregnant individuals that cfRNA transcripts in maternal blood predicted gestational age with comparable accuracy to ultrasound, and in 38 pregnant individuals, seven cfRNA transcripts in maternal blood could predict preterm birth up to 2 months prior to preterm delivery. This manuscript also builds upon findings described in a s2014 manuscript demonstrating the potential of cfRNA in maternal circulation to monitor fetal-specific tissue transcripts (largely originating from placenta; Koh et al, PNAS, 2014).

Validity: no concerns, robust study design with large validation cohort

Originality: The large cohort size and substantial racial and ethnic diversity are strengths of the study, but the concepts of determining gestational age via cfRNA in maternal blood, monitoring fetal development via cfRNA in maternal blood, and early biomarkers for later-onset preeclampsia are at this point not novel in and of themselves (reported in prior publications as cited above). Numerous other studies have investigated the test characteristics of early biomarkers/predictive markers for later development of preeclampsia, including gene expression signatures and other omics approaches (there are numerous, just a few examples: Banadakoppa et al, Sci Reports, 2020; Tarca et al, J Maternal-Fetal Neonat Med, 2019; Odibo et al, Prenat Diagn, 2011; one even by one of the same authors as this paper- Yadama et al, Sci Reports, 2020).

Comment #1: We appreciate the cited research and value what that body of literature has brought to the field, these studies (and others) serve as excellent proof of principle studies. It should be noted that these studies each have total sample size of less than 200, are across limited racial and ethnic diversity and typically draw on a single cohort.

Importantly to parlay the scientific power of cfRNA, the known variability of gene expression among human subjects, and to be able to do so from a single blood draw –mandates large diverse data sets. As such, this study is novel, as findings such as independence from clinical factors can only be found in large sample sizes. Our preeclampsia prediction works across two cohorts and with a larger sample size than any prior published model of preeclampsia – thus taking this platform beyond proof-of-principle.

The positive predictive value of 32.3% for preeclampsia is not incredibly high (FFN for predicting preterm birth has a PPV ranging from 17-30% depending on studies, by comparison), and likely the PPV would be slightly lower in other cohorts with a more typical occurrence of preeclampsia (e.g. 10% versus 13%). Thus, it's not clear that this represents any sea-change in the monitoring for or care of pregnancies at risk for preeclampsia.

Comment #2: We appreciate the reviewers focus on this important metric. For screening tests, it is important that PPV be considered in the context of the subsequent intervention. As examples, we compared our findings to two widely adopted screening tests:

1) Colon cancer screening (Cologuard) has a PPV of 4.6% (Imperiale et al, NEJM, 2014) and this is accepted since the cost of Colonoscopy as the immediate next step is worth the higher cost of missing someone with a life-threatening condition.
2) Screening Mammography has a PPV of 5% (Lehman et al, Radiology, 2017) and this is accepted since the cost of a Diagnostic Mammography as the immediate next step is worth the higher cost of missing someone with a life-threatening condition.

With that context, if we can then view the effectiveness of screening tests in the OB-GYN space. For *preterm birth* newer tests (e.g. Sera PreTRM) are designed to predict months in advance – enabling therapeutic intervention – however their PPV is 2-7% (Farnsworth et al, J Appl Lab Med, 2021). For *preeclampsia* the state of the art for screening is PPV 4.4% (Table 4, Tan et al 2018). Our test offers a >7-fold improvement with a PPV of 32.3% and with 14.5 weeks advance notice – a paradigm shifting opportunity for therapeutic intervention – and the cost of the immediate next step (LDA low-dose aspirin and home BP monitoring) is certainly worth the much higher cost of missing someone with a life-threatening condition like Preeclampsia.

FFN is a biomarker test used on cervicovaginal fluids to assess a woman's risk of preterm birth. Its clinical utility is limited to women who **have symptoms** of preterm labor, and it is largely used as a rule-out test. Its predictive value is less than 10 days from the test is administered. This type of biomarker test must be distinguished from a screening test run in **asymptomatic** individuals months prior to onset of any symptoms.

Data and Methodology:

1) Page 5, lines 183-207: The term "fetal organ signal" is imprecise and doesn't address some key questions a reader is left with after the manuscript: (1) if these transcripts truly reflect fetal organ development and not just the placenta, then how might these fetal organ-specific transcripts be getting into maternal blood, and (2) how can the authors be sure these transcripts aren't just reflecting apoptosis of a specialized set of placental cells, rather than true fetal organ development. CfRNA in maternal blood has been demonstrated to largely originate from the placenta/placental apoptosis, even in a manuscript by some of the same authors in 2014. Placental-origin transcripts also would be expected to increase progressively across gestation, so that aspect is not particularly convincing that these transcripts are shedding directly off fetal organs. In addition, maternal cardiac and renal transcripts also could certainly be changing across gestation, so more detail is needed on how these transcripts are known to reflect the development of fetal organs rather than maternal or placental. Some more discussion of the origin and trafficking of cf fetal RNA (cffRNA) in maternal circulation is warranted.

Comment #3: Based on feedback from these reviews we have modified our analyses on fetal development by adding more supporting evidence and additionally new data has become available expanding the number of fetal data sets we could compare against. Firstly, we show the expected change found from gestational age model genes and placental genes, which as the reviewer suggests are increasing as a function of pregnancy progression. From analyses of fetal gene sets we exclude genes from placenta or the gestational age model to avoid confounding of the fetal gene set signatures. We observe a total of 40 fetal gene sets that increase and 15 that decrease with gestational age. We added full list of gene sets (fetal and otherwise) that change with gestation in a supplementary file.

2) Do any of the participants included here have overlap with participants included in other publications by some members of the same group (e.g. the Science 2018 paper)? Or are these all new participants?

Comment #4: These are all new participants from different cohorts

3) Page 5, lines 183-190: Are all of the organ-specific transcript results based on 95 women? While the text mentions N=95 women who had data available for all 4 collections, the figure mentions N=91, this should be reconciled. Were the additional 3 cohorts A, B, G also used to try to discover more organ-specific transcripts? Or only to verify increase of the organ-specific transcripts identified in the original 95 across gestation?

Comment #5: We have corrected the numbers to match 95 throughout the text. Gene sets were discovered in cohort H where 4 longitudinal samples were available and confirmed in the three independent cohorts where at least two draws were available.

4) It's interesting if fetal organ transcripts are detectable in maternal circulation that there would be no gestational-age-specific or evolving pulmonary or brain signal across gestation, as those organs are known to continue to mature significantly across gestation. Why do the authors think there was no signal from other organs that would be of significant interest with respect to non-invasive monitoring of their maturation/function?

Comment #6: The gene sets depicted in figure 2 are only a small set of all fetal sets where we observe a change, full list is now included as a supplementary table/file and we have depicted gene sets related to placenta, fetal (developing heart) and maternal (collagen/extracellular matrix) to illustrate that pregnancy changes in a predictable manner.

5) Functional analyses of the differentially-expressed genes across gestation that are reportedly reflecting small intestine, renal and cardiac development would enrich the manuscript and provide potentially important insights/increase significance.

Comment #7: While we agree functional analyses would be interesting it is not within the scope of this study.

6) Page 5, line 218 on- discussion of predictive test for preeclampsia in 2nd trimester blood:
Several points merit clarification/additional information

a) Knowledge of when the participants developed preeclampsia relative to when the 2nd trimester blood sample was taken is very relevant to gauging how much of an advance this test could be, and how it differs substantively from other 3rd trimester tests which detect preeclampsia very close to when clinical features would declare themselves. Women can certainly develop preeclampsia at 27 weeks and close to 27 weeks, so a supplemental figure depicting when each of the preeclampsia participants had blood drawn versus when they developed preeclampsia would be very useful, as well as median (IQR) for time from blood draw to development of preeclampsia (this could be in Table 1). It would also be helpful if Table 1 showed how many of the preeclampsia cases and controls came from which cohort.

Comment #8: We have added Figure 3a to help illustrate the distribution of sample collection and delivery times. Although we don't have data for when the women develop preeclampsia, on average the women deliver 14.5 weeks after blood draw, further supporting they were asymptomatic at the time of blood draw. We have added additional information on cohorts and samples in supplementary table

b) The use of an old definition of preeclampsia (based on 2013 rather than updated 2017 definitions) is potentially problematic. Why was this older definition used? If participants were properly classified using up to date preeclampsia definition, would the results hold? Would any of the "gestational hypertension" control cases have met the 2017 definition for preeclampsia by lab abnormalities?

Comment #9: Case definitions (GHTN/PE) were determined by the PI of the primary cohorts. We did not have information to adjudicated severity of PE based on prior or current ACOG definitions (e.g. abnormal liver enzyme tests). Additionally, there is increasing evidence that it is the gestational age of occurrence rather than the details of diagnostic criteria that mark prospective differences in PE pathophysiology (Myatt & Roberts, Curr Hypertens Rep 2015; Rana et al, Hypertens Preg, 2013; Leavey et al, Hypertension, 2016; Redman & Sargent, Science 2005).

c) Given that at least 3 of the 7 preeclampsia RNA markers are related to embryo implantation, early miscarriage, and early placental development (CLDN7, TLE6, PAPPA2), it seems to make sense to see how the panel of 7 performs for predicting preeclampsia from 1st trimester blood draws. Figure 1 suggests there are

plenty of first trimester samples, so there must be a reasonable number of preeclampsia cases who have first trimester samples to test. Is there a reason this evaluation of marker robustness for an earlier timepoint in pregnancy (potentially at an intervenable period with baby aspirin initiation) was not performed?

Comment #10: The samples collected across pregnancy are primarily from healthy pregnancies and we do not have sufficient first trimester samples diagnosed with preeclampsia to evaluate robustness of our markers at an earlier time point. This would be an interesting follow up study if sufficient samples from first trimester can be sourced.

d) What were the 452 non-cases of preeclampsia "matched" to the preeclampsia cases on the basis of? 452 is not a multiplier of 72, so unclear how the matching was performed and details are not provided.

Comment #11: Samples were matched based on gestational age at blood draw, ethnicity, BMI, smoking status and maternal age, we have added a description of matching to our supplementary text in the cohort section. Our study is done largely as a case-cohort, although non-cases are enriched for preterm deliveries (n=89), and we did some matching of non-case population as described above.

e) Page 7, lines 267-270: "both aligned well with what is known about preeclampsia pathophysiology" is too general of a statement. How does downregulated gene sets related to immune pathways align with what is known about preeclampsia specifically, for example?

Comment #12: We have modified the text, it now reads "Both the upregulated and downregulated gene sets align with the accepted primary pathogenesis of PE" this aligns with established literature as reviewed by Redman & Sargent (Science 2005)

f) The discussion of the 7 genes that predict preeclampsia should include how this set of 7 genes overlaps or doesn't overlap with lists generated by Munchel et al (7) or Moufarraj et al (9).

Comment #13: Given the very different collection windows and the dynamic nature of transcription levels, comparing sets across the three studies would not have a clear expectation. Running the analyses shows no gene overlap between Moufarraj and the two other studies, and a single gene overlap (PAPPA-2) between Muchel et al and our study.

Appropriate use of statistics and treatment of uncertainties: No concerns

Conclusions: As referenced above, the utility and novelty of this second trimester test for preeclampsia is somewhat difficult to gauge, given the relatively low PPV, use of outdated definitions of preeclampsia, and lack of information about how long the blood was drawn before clinical symptoms manifested. Given the low PPV, the statement "a cfRNA platform enables early detection of multiple clinically relevant endpoints (e.g. gestational age and preeclampsia) from a single specimen…" (lines 327-28) is overstated. The strongest evidence offered in the paper is a confirmation/validation of the 2018 paper in Science demonstrating relatively robust prediction of gestational age +/- 2 weeks from cfRNA in a maternal blood sample. The evidence that cfRNA can be used to predict preeclampsia in a meaningful way and to monitor fetal organ development is less robust, for the reasons detailed above.

Comment #14: We appreciate the feedback from the reviewer, we have updated our manuscript to be clearer in various areas pointed out by the reviewer. Regarding PPV and the potential utility of the test we have addressed this in detail in comment #2

Suggested improvements: would be performed in response to concerns raised above.

References: no concerns

Clarity and context: clearly written, appropriate abstract, some of conclusions read as overstated as previously described.

Referee #2 (Remarks to the Author):

The goal of this study was to demonstrate the utility of cell free signatures in 3 contexts: (1) gestational age estimation (2) monitoring fetal organ development and (3) as a biomarker for preeclampsia. Data was collected across eight different cohorts in over 1840 participants, representing a very large sample size. Overall, the researchers are using a very novel method to tackle a number of challenging problems in pregnancy. The blood test for gestational length has broad applicability, particularly in low resource settings. Each concept alone would be very valuable, but by grouping all three analyses together, a lot of key details are missing, and analyses are presented in a very shallow way.

The supplemental methods section is really hard to follow, as it doesn't follow a logical progression, and is missing a lot of details. Some parts of the statistical analyses seem valid (such as the cell free RNA data generation, and the gene set enrichment analyses), but without more detail about the underlying data, it is hard to understand how much these findings are impacted by batch effects.

Comment #15: Based on reviewer feedback we have added additional information to the supplement and structured it so it follows chronologically where it is referenced in the main text to make it easier for the reader to find information.

Moreover, one of the biggest strengths of this paper is the strong ethnic and racial diversity of the cohort, but this data isn't analyzed or presented appropriately.

Comment #16: Please see discussion under comment #28 below.

Part 1: Cohorts Involved

1. The authors provide limited information on the cohorts in table 1, with more detailed information provided in the supplemental materials. I find the presentation of cohorts A-H very confusing, and would prefer if the co-authors just referred to the individual cohorts by name or acronym, or at least presented this information in part in supplemental table 1.

Comment #17: We chose the A-H labeling to assist the reader. We have added supplementary table with additional cohort information.

2. This manuscript is using a number of different cohorts that are from very different parts of the world, and with data collected at very different timepoints, and with very different goals. It would be helpful to clarify this in the manuscript text, and in table one to clarify the dates of cohort collection and/or if collection is still ongoing. This is relevant because some of these studies span from 1998 to the present day, and there are a number of fundamental changes that have occurred in prenatal care and the treatment of gestational diseases within this timespan.

Comment #18: Good point, we will add collection time range to supplementary table with cohort information

3. The authors present the exclusion/inclusion criteria for each study individually. However, was there a unifying set of inclusion/exclusion criteria beyond each study that was used for the paper itself. Or any sub-analyses? For example, for measurements of gestational length, I would exclude all multi-fetal pregnancies due to inherent differences in gestational length in these pregnancies.

Comment #19: For the gestational age modeling we used healthy pregnancies and throughout the study we only included singletons. We added additional text to the supplementary materials to better explain both sample usage and cohort structure.

Overall, the clarity of this manuscript could be improved by adding a supplemental figure/table to the paper that follows the STROBE guidelines through a STROBE diagram.

Comment #20: Given the very diverse nature of the cohorts following strict guidelines such as STROBE is very hard to implement. We have added a section in the supplement addressing this.

4. One of the largest strengths that the authors highlight is the diversity of the cohorts, however this is not true for all datasets. For example-Dataset H was collected in Denmark and is almost entirely white, with no other races presented. This is significant because The molecular signatures for fetal organ development only uses data from cohort H (Line 187).

Comment #21: It is correct that the molecular signatures are discovered in the Danish cohort, but all signatures are verified in three other cohorts, two from the US and one from Africa. We have added data to figure 2 to illustrate this point (panels 2i-l). The diversity in the data set is achieved by combining cohorts from across the globe, even if individual cohorts by themselves are not diverse.

5. The authors group race and ethnicity together in their descriptions and analyses in Table 1. Race and Ethnicity are 2 separate constructs and should be presented independently. The authors could also stratify into something like-black Hispanic, white Hispanic, white-non Hispanic, black non Hispanic as an alternative.

Comment #22: We have clarified our language and will only address race as numbers for individual ethnicities are insufficient.

Part 2: General RNA sequencing Comments

6. Since the authors are combining data from so many cohorts and timepoints, there are bound to be substantial batch effects. The authors mention in their study design that each cohort was treated as an independent batch and a batch correction was applied prior to modeling the data. (Line 117) However, there is no mention off this in the supplemental data at all.

Comment #23: We have a section (Feature normalization) in the supplement on cohort correction, which explains our approach to correcting for variation introduced by each cohort. We adjusted title to clarify what is covered.

The authors discuss feature normalization, but never present any effects of batch on results. There is one figure (line 686) that has batch effects for just one gene, but there should be some sort of PCA plot showing batch effects across all genes, as they have done to show a lack of variation associated with the data to investigate

how covariates influenced gestational length and preeclampsia . Also, in this plot they mention that each cohort has its own color but the cohorts aren't labeled and the choice of color is not optimal for categorical data. I am also wondering if the authors performed separate batch normalizations before or after processing their data.

Comment #24: Supplementary section describing this has been clarified. The single gene plot is illustrative as we ran our correction on a per gene basis. All corrections were done independently for train and test sets, to avoid introducing false signal.

7. Along the lines of batch effects, it seems like there are layers of batch effects present here. For example, the GAPPS biorepository collects from 2 different locations, introducing batch effects at the cohort level. Moreover, with this many samples could not have been analyzed in one RNA sequencing run, so there also must be batch effects within the sequencing runs.

Comment #25: Batch effect can exist at many levels and fully correcting for it is not possible. However, in the analyses using all 8 cohorts splitting samples in to train and test sets prior to performing any corrections should alleviate any major impact from batches.

Part 2: Molecular signature of Gestational Age

8. The wording of this "gestational age" is very confusing. Are the authors predicting gestational age at the timepoint of sample collection or at the timepoint of delivery. In the abstract and at some parts in the manuscript, they simply refer to gestational age, but at other points they refer to gestational age progression (line 96). Adding a sentence and tightening up the language could clarify this

Comment #26: We tried to clean our language throughout, we are predicting gestational age at time of blood draw.

9 .The authors strategies of performing cross validation in the training dataset and then testing in the test dataset for lasso regression are valid. Can the authors show the distribution of sample information (as in table 1) for the training and testing set? What cohorts were the samples taken from?

Comment #27: Samples were randomly split across each cohort with 80% going to the training set and 20% to the test set. We have clarified this in the main text.

10. It is hard to understand how the clinical factors could impact the model construction without knowing what cohorts the model was constructed from. The authors state that inclusion of clinical factors did not improve the model much, which is surprising. The authors only include maternal BMI, maternal age, and maternal race/ethnicity. However, these list of covariates (for this analysis and others) are not inclusive of all factors that might impact gestational length. I am surprised the authors don't have a metric for maternal socio-economic status, such as maternal education or maternal income. I understand it would be challenging to come up with a harmonized variable across cohorts, but without a marker of SES it hard to interpret.

Comment #28: For gestational age, the model is constructed across all cohorts, for preeclampsia we used cohort A and E. The reviewer correctly points out that social and demographic variables are associated with a higher or lower risk for pregnancy outcomes such as PE. What is so unique about these data are that they speak to the underlying biology. Thus, regardless of risk factor (exposure to air pollution, violence, racism, etc.) that the molecular effect (e.g. disruption of trophoblast invasion) is a conserved mechanism that can be revealed by our cfRNA profile. The major driving clinical factors associated with an increased in PE are BMI and race.

Our data (figure 1) demonstrate that these do not alter the cfRNA profile—as we would expect since it is revealing not risk but biology.

11. In the table presented (line 707), what does pctvarexp stand for? I am presuming it is percent variation explained, but this should be spelled out (as well as for all presented on here).

Comment #29: Yes, percent variance explained. We have updated the figure legend.

12. It it would be useful to present in a supplemental table what the features that best predicted gestational length are, or (if this is proprietary) the authors could provide some insight into the biological functions.

Comment #30: Absolutely, we have added a supplementary file with all genes used in the gestational age model and the importance (weight) of each gene in the model.

Molecular Signatures of Fetal Organ Development

13. Supplemental Figure S1 does a nice job of showing trends in key pregnancy endocrine pathways across pregnancy. However, the Y axis does not make sense because they are all the same number, likely due to a plotting issue.

14. What exactly do the authors mean when they say "analyzing gene sets and by targeting gene sets" (line 185). How were these gene sets defined? It seems odd to me to use GO pathways to do this, when there are databases of tissue specific genes. However, in supplemental Table S1 they list a number of papers. How were these papers selected?

15. How are the authors defining significant changes in gene sets (up or down) (Lines 214-216).

16. Supplemental Figure S1-Again the axes here do not really make sense. Additionally, if the authors were to restrict to a smaller window of space on the Y axis, it would be easier to see changes.

Comment #31: Ad 13-16. We changed our section on gene set analyses including the figures, see comment #3.

Classifying risk of preeclampsia

18. What does NES stand for in supplemental figure S4. Also, please check the spelling of the titles. Moreover, the authors only present the top 20 genes, but since they are mentioning 88 significant fetal gene sets (Line 738), it would be helpful to present the full results in a supplemental table.

Comment #32: NES is normalized enrichment score, we have made sure it is clear from the figure legend. This analysis is done using gene sets selected from the gene ontology set and are defined by functional groupings, this is different from the fetal gene sets that are defined based on single cell analyses.

19. Line 226: what was the criteria for matching? Were there a matched % of preterm birth samples? How was the data distributed throughout the cohort? A table here showing this data would be helpful.

Comment #33: We have added text to the supplement to address this point. See also comment #11.

20. The authors perform an ANOVA on a very limited number of covariates and claim that they do not influence risk of preeclampsia. This is pretty surprising, given the breadth of studies showing that Black women at are at higher risk of preeclampsia. A more in depth analysis would be required, including reporting the number of cell free RNA transcripts associated with maternal race and ethnicity alone, and a description of how batch effects were handled. The authors should also consider adjusting for other confounders associated with increased risk of preeclampsia, such as a history of preeclampsia, gravidity.

Comment #34: As with our gestational age model, cfRNA alone can explain the data and adding clinical factors does not improve the model performance. This would indicate that the pathophysiology our model identifies is independent of clinical factors including race. We agree with the reviewer that Black women are at higher risk for PE. However, we disagree that this is a genetically mediated event. As such, we would not expect, nor did we find, a change in cfRNA abundance by race. Contemporaneous thought with a more holistic view including the biological effects of racism support social determinants of health in elevating risk of PE for Black women (Vyas et al, N Engl J Med 2020). As such, we hypothesized that the cfRNA should reflect conserved biology of disease. Our data prove our hypothesis to be correct.

21. How does this model of preeclampsia compare to other models of preeclampsia using omics data?

Comment #35: We have added some discussion of published models of PE

Discussion:
22. It remains unclear if an ANOVA and correlation of the first 2 PCs alone is enough to conclude that there is not confounding by race/ethnicity, as the authors claim (Line 296). I think this sort of conclusion could only be made if the models were run with and without these covariates.

Comment #36: We did add the clinical factors as covariates in the analyses and they only explain a small fraction of the variance in the data.

23. It seems odd to me to group these 3 separate analyses in one paper, and not compare and contrast or build the cell-free RNA signatures against each other into a cohesive story.

Comment #37: We have contextualized our choice and updated the text to make the story more coherent. GA model and gene set analyses serve as examples of pregnancy physiology as described by cfRNA, while preeclampsia serves as example of a pathophysiology

24. This manuscript is hard to follow, but more specific titles, both in the figures and in the headings of the supplemental text, would make things more clear

Comment #38: We updated the main text, figure legends and supplementary materials to clarify

Referee #3 (Remarks to the Author):

Rasmussen et al have expanded on their earlier work to further explore the potential clinical value of cfRNA analysis in maternal blood. Three pregnancy-related facets have been investigated in this study:
1. the feasibility to estimate the gestational age by cfRNA analysis
2. Whether cfDNA profile would offer information regarding fetal development
3. cfRNA analysis as predictive markers of preeclampsia

These investigations were performed on samples dervied from 8 recruitment cohorts. Because all the analyses have been performed retrospectively in batch formats and no prospective interventions had been applied to any of the cohorts, describing the samples as having been prospectively collected in the manner shown in lines 107 to 109 is somewhat misleading.

Comment #39: Samples for all 8 cohorts were collected prospectively. We analyzed them in a case-cohort design after collection.

Regarding the facet on gestational age prediction, the authors have been able to improve on their previous work published as Ngo et al Science 2018. In Ngo et al, gestational age estimation for each pregnancy was performed using the levels of certain cfRNA transcripts taken across several dates. In the present work, the authors achieved gestational age estimation at errors within ~ 15 days with the use of just one blood sample. By performing the feature mining on a racially and ethnically diverse sample set, the authors showed that the resultant metric was mostly dependent on gestational age. Other demographic factors contributed to < 1% variance.

The conclusions drawn for this part of the study are robust which is contributed by having a subset of IVF pregnancies to verify the accuracy of the gestational age prediction; a relatively large sample size; segregating the sample cohort into training and validation sets; as well as checking for evidence to exclude the possibility of over-fitting.

However, one interpretation of the data has been overstated. In lines 176 to 177, the authors seem to be forecasting the utility of the gestational age prediction algorithm to contribute to the clinical management of small-for-gestational-age fetuses. This is too much of a leap of faith because the dataset did not seem to have included such pregnancies. It is not known if the prediction algorithm would work equally well in such pregnancies whereby the suboptimal fetal growth might be due to underlying pathologies that could alter the cfRNA profiles.

Comment #40: We agree and have removed this statement.

Regarding the second facet of the study, gene sets considered by gene ontology to be associated with fetal small intestine and developing heart were observed to increase with gestational age advancement while the gene set identified as associated with nephron progenitor was observed to reduce with progressive gestational age. Based on these gestational-age related trends, the authors interpreted the data as providing a window into fetal development (line 216). It is somewhat premature to make this conclusion when the associations of the sets of cfRNA transcripts with fetal intestinal / heart / kidney development have not been verfied using samples with fetuses showing developmental issues in those organs. Some of the authors have also reported the detection of "fetal brain" transcripts in maternal plasma in Koh et al Proc Natl Acad Sci U S A 2014.

Comment #41: We have updated this analysis to include changes observed in placental, fetal and maternal gene sets. True origin is impossible to determine, but changes are significantly correlated with gene sets defined from single cell analyses from each tissue.

Furthermore, the data mining was performed on timed serial samples collected from 95 women as part of cohort H which according to Figure 1 is a homogenous population of one ethnic/racial group. This is a much smaller and much less diverse sample set than what the authors have described their study design as more generalizable due to the sample size and diverse populations (lines 106-107).

Comment #42: While the gene sets are discovered in cohort H, they are verified in three other cohorts to address the issue of diversity. We have added this information to the main text figure panels 2 i-l.

The third part of the study involved 72 cases of preeclampsia and 452 controls from cohorts A and E. Using a panel of cfRNA transcripts, a sensitivity of 75% and a PPV of 32.3% were achieved. It would be useful to know and stratify the preeclampsia cases into the commonly used clinical phenotypes, namely late vs early onset, severity and if asprin has been administered early in gestation.

Comment #43: We have added a graphical representation of sample collection and delivery (figure 3a). To have sufficient samples to run meaningful statistical analyses all preeclampsia cases were grouped, and the classifier evaluates at the gross level, numbers are not sufficient to stratify further.

With or without the stratification, having 72 cases of preeclampsia still falls within the tens of cases the authors considered as limitations of the prior studies (line 300).

Comment #44: We have removed this statement as part of the rewrite, although we were referring to total study size not case count in the specific sentence.

In fact, in the first two paragraphs of the discussion, the key advance of the present study was identified as the large sample size and broad diversity of the study cohorts. Yet, this advantage mainly rests with the first facet of the study and less so for the other two parts.

In summary, cfRNA analysis may offer much potential for monitoring pregnancy health. The present study is definitely a step in the right direction. The manuscript is succinctly written with the data and methods clearly presented. However, there are quite a number of overstatements, priority or unqualified statements. For example, the advantage of the size and composition of the sample cohorts have been too broadly generalized to all facets of the study. Another example of a unqualified statement is that claim in lines 83 to 85 indicating an early cfRNA test would contribute to advancements in fetal maternal health globally. Nothing in the present manuscript is clinic-ready. The manuscript title is also unjustified suggesting the work offers a molecular window into maternal fetal health. The only part that may be relevant to "maternal health" is perhaps the mentioning of preeclampsia might put the mother at lifetime risk of cadiovascular consequences. No data in the manuscript showed that one could prognosticate the mother or rule out maternal disease.

Comment #45: We have rewritten large parts of the main text and been careful to avoid overstating our results.

The authors seemed to suggest what has been observed in the use of liquid biopsy for breast cancer could predict the clinical value of pregnancy cfRNA tests (lines 338 to 340). As for "fetal health", there is not enough evidence in the manuscript to show clinically actionable differences to ensure or maintain fetal health using the cfRNA data.

Reviewer Reports on the First Revision:

Referee #1 (Remarks to the Author):

While the authors have addressed some concerns, the manuscript does not differ substantively from the original submission. The manuscript could be strengthened with some additional data, and/or discussion as below.

1. Manuscript still lacks discussion of the biological plausibility of how cfRNA transcripts that are fetal rather than placental would make their way into maternal circulation, and how it has been determined that specific transcripts reflect fetal organs rather than the placenta or maternal cfRNA, when placental and maternal cfRNA would certainly be expected to dominate transcripts in maternal blood. While the authors state they added additional "fetal gene sets" (assume based on information provided this means existing gene sets used as comparators for their data) that increase and decrease with gestational age, the Supplemental Data Set 2 appears to be just a list of comparator resources and the actual "fetal gene sets" that increase or decrease with GA in this study (the 40 up and 15 down that are referred to) are not detailed anywhere I can find (e.g. the component genes of these 55 gene sets that are altered by gestational age and reflect fetal development, as framed in the paper). A discussion of how transcripts from fetal organs might make their way into maternal blood is still lacking. As putative "placental", "fetal cardiac", and "maternal collagen-ECM" genes all increase across gestation in relatively similar patterns, more discussion of the 15 gene sets that decrease with gestational age and what these reflect about fetal development might also be helpful here.
The claim to have shown "the first non-invasive window into maternal-fetal development from a maternal liquid biopsy sample" and the comparison with breast cancer liquid biopsy is overstated based on the data and analyses presented for this part of the paper. As one of three main foci of the paper is the claim that cfRNA can reflect fetal development via organ-specific transcripts, functional analysis (e.g. with GO) to show how sets of genes that increase and decrease across gestation provide insight into fetal biology is within the scope of the paper. Currently this part of the manuscript could be improved with more substance.
A minor comment about this aspect of the paper is that Figure 2i-l needs to explain the meaning of the colors used in these panels.

2. The authors have presented a compelling argument for why a test with PPV in the range of 32% has utility. Assuming that this test is appropriately robust and useful for detecting preeclampsia, the manuscript still needs to detail a more compelling rationale for how detecting preeclampsia risk up to 14.5 weeks in advance would change management or disease course. A basic tenet of screening that a positive screen offers the potential to change the disease trajectory or outcome. The authors themselves state the rationale for earlier diagnosis as identifying those at risk early in pregnancy when there is a "window for preventive strategies." It is not clear that a positive second trimester screen for preeclampsia has that utility. The rationale offered in the response to reviewer comments (to start low dose aspirin and home BP monitoring) is somewhat thin. Low-dose aspirin started much after 12 weeks and certainly after 20 weeks is unlikely to alter the trajectory of preeclampsia, based on biological plausibility of how it might work to impact trophoblast invasion/placental function. While the mechanisms of LDA for preeclampsia prevention remain unclear, it does seem clear that starting it after 20 weeks for this purpose has little impact. Figure 3a suggests the majority of samples were drawn around 18-20 weeks and with another peak around 24-25 weeks, both of which seem late to start LDA to impact placental function. In addition, if home BP monitoring is the intervention after the screening test, some evidence demonstrating efficacy of home BP monitoring in altering the trajectory of preeclampsia and/or improving maternal outcomes in cohorts at high risk for preeclampsia should be offered in the manuscript's discussion to frame why the screening test with 14 + week lead time will be impactful in translation/why such a test is needed.
The ability of the test to predict preeclampsia in the first trimester might lend some biological utility, as the first trimester is a time when starting LDA actually might alter disease course, but

the authors state there aren't enough patients in the cohort with first trimester blood draw who had preeclampsia to assess the utility of their predictive test in this setting. To truly demonstrate why this paper represents a "paradigm shift" in preeclampsia prediction, as the authors state in response to reviewer comments, more rationale/explanation must be provided as to why/how such a test will alter disease trajectory or maternal outcomes. One use of the test in real-world settings could be to delineate preeclampsia from an exacerbation of chronic hypertension, as the authors describe, but this is not using the test to "predict" preeclampsia, which is the main focus of the paper.

3. Similarly, more framing of how the advance of dating a pregnancy to the same accuracy as second trimester ultrasound is practice-altering or field-altering would be informative and strengthen the paper. Do the authors feel this would be potentially used in low-resource settings? In settings with limited prenatal care? A brief statement highlighting the relevance would be useful.

4. There is still insufficient detail provided in the main text about how the case-control study was conducted in cohorts A and E. How/based on what criteria were the 72 "cases" of preeclampsia matched to 452 "non-cases"? For example, the rationale for matching gestational hypertension as a "non-case"/control is unclear. Gestational hypertension has been increasingly recognized as on the spectrum of preeclampsia and has similar implications for maternal health and pregnancy management, so wouldn't be an appropriate "control" against which to match preeclampsia samples for a prediction model. It might be worthwhile to examine what happens if these 19 cases from cohort E were grouped with the preeclampsia cases instead, as both are thought to be a "placental disorder" in contrast to chronic hypertension (e.g. page 8 line 253). More detail should be provided about the "case-control" design here as preeclampsia prediction appears to be the main feature of the paper as currently written.

6. The demonstration that the biology is independent of race in preeclampsia is useful and the authors should be commended for framing their results in this manner.

Referee #2 (Remarks to the Author):

The authors have revised their manuscript and thoughtfully addressed reviewer comments raised by myself and others. Below, I have a few additional questions that were not clarified for some specific comments:

1. The authors addressed Reviewer 2 comment 18 to add the timepoint of sample collection, but do not address the original comment: "This manuscript is using a number of different cohorts that are from very different parts of the world, and with data collected at very different timepoints, and with very different goals. It would be helpful to clarify this in the manuscript text, and in table one to clarify the dates of cohort collection and/or if collection is still ongoing. This is relevant because some of these studies span from 1998 to the present day, and there are a number of fundamental changes that have occurred in prenatal care and the treatment of gestational diseases within this timespan." I do not see the date range when the samples were included (For example, the POUCH study was collected from 1998-2004, but the other studies do not have this information listed) in either Table 1 or Supplemental Table 1. This is also not mentioned in the manuscript text as a concern.

2. I am still confused about the presentation of race/ethnicity in table 1, where Hispanic/non Hispanic (ethnicity) should be separated from black, Asian, white (Race). Otherwise, it looks like the authors addressed this.

3. In response to reviewer comments (from myself and others) about the challenges of presenting

the data from only cohort H in the model, the authors add panels I-L to demonstrate signal across all cohorts with longitudinal data. However, there are only 4 lines here and it is not clear what color corresponds to which cohort. Can the authors clarify this picture.

Referee #3 (Remarks to the Author):

The authors have adequately addressed my prior comments. I have no further comments on the manuscript and study.

Author Rebuttal to First Revision:

Referee #1 (Remarks to the Author):

While the authors have addressed some concerns, the manuscript does not differ substantively from the original submission. The manuscript could be strengthened with some additional data, and/or discussion as below.

1. Manuscript still lacks discussion of the biological plausibility of how cfRNA transcripts that are fetal rather than placental would make their way into maternal circulation, and how it has been determined that specific transcripts reflect fetal organs rather than the placenta or maternal cfRNA, when placental and maternal cfRNA would certainly be expected to dominate transcripts in maternal blood.

Comment #1:

While mechanisms of transfer remain to be elucidated, it is well established that fetal cells and the components of fetal cells are present in maternal circulation. A widely cited example of this fetal-maternal crossing is Dr Bianchi's work showing the presence of Y-chromosome containing CD34+ cells isolated from maternal blood in pregnancies with male offspring (expanded study: Khosrotehrani et al, JAMA, 2004). Other examples include the highly regulated maternal immune response with development of fetal antigen-specific maternal T-regulatory cells (Kahn and Baltimore, PNAS, 2010) or the presence of fetal neuronal exosomes in maternal plasma (Goetzl et al, 2016, Annals of Clinical and Translational Neurology). We agree with the referee that maternal and placental RNA far outnumber the fetal contribution. Given the known leakage of cellular material from fetus into maternal blood stream, identifying the presence of fetal cfRNA is not unexpected especially with an assay optimized for sensitivity.

Understanding that there are very few transcripts expressed that are truly unique to fetal cells, we utilized previously established fetal genes sets to validate the fetal cfRNA profile. To address this comment and demonstrate signal from fetal genes, we isolated genes across all the fetal gene sets that are not found in any adult gene set or gene ontology categories or in placenta. The removal of these genes resulted in 78 genes that are of 'exclusively' fetal origin, we observed counts in our data for all 78 fetal genes.

While we observe these individual fetal gene transcripts, tying them to a biological process is not straight-forward. To show a more robust link to the underlying biology, we utilized established fetal gene sets discovered in single cells isolated from fetuses, and showed that the cfRNA counts from several genes across the gene set, all move together. We identify a number of specific examples where the gene sets we observe show similar cfRNA patterns to what was observed in similar gestational ages in fetal single cell studies. Please see further details in comment #3.

We have added references substantiating the presence of fetal material in maternal circulation. Additionally, we have incorporated the analyses on pair-wise comparisons of time points in Cohort H into the supplement.

While the authors state they added additional "fetal gene sets" (assume based on information provided this means existing gene sets used as comparators for their data) that increase and decrease with gestational age, the Supplemental Data Set 2 appears to be just a list of comparator resources and the actual "fetal gene sets" that increase or decrease with GA in this study (the 40 up and 15 down that are referred to) are not detailed anywhere I can find (e.g. the component genes of these 55 gene sets that are altered by gestational age and reflect fetal development, as framed in the paper).

Comment #2:
We appreciate the reviewer drawing our attention to how our text was not clear. The fetal gene sets, described in the paper, are defined from published single cell RNAseq from fetal samples as noted in response above. We do not define any new gene sets from our data, rather we observe these pre-defined gene sets in cfRNA and track them longitudinally over the course of pregnancy. Consistent with expected fetal development, some of these genes sets increase over gestation while others decrease. Having a group of genes all changing as prescribed in an independently defined set is strong evidence of their cellular origins.

We have clarified our language in the main text to more clearly state that we confirmed changes in published gene sets. To help clarify which genes are used in the fetal gene sets, we have extracted the gene names from the sets in supplementary data set 2 and added them as individual sheets in the excel workbook.

A discussion of how transcripts from fetal organs might make their way into maternal blood is still lacking. As putative "placental", "fetal cardiac", and "maternal collagen-ECM" genes all increase across gestation in relatively similar patterns, more discussion of the 15 gene sets that decrease with gestational age and what these reflect about fetal development might also be helpful here.

Comment #3:
Additional data are now provided that demonstrate fetal gene sets that either decrease or increase across gestational age as would be expected from what is known regarding specific fetal organ development. We show nephron progenitor cells as an example of a decreasing gene set, which aligns with the decreasing nephrogenic zone width as a function of gestational age. We also included two gene sets from development of the gastro-intestinal tract, 1) esophagus illustrating early development and a drop over gestational age and 2) small intestine which develops later and has strong increasing expression over gestational age. These results independently confirm the findings from the single cell study (Gao et al, Nature Struct Biology, 2018), and thus illustrates how a liquid biopsy can give a window into fetal development.

We have incorporated the analyses of additional fetal gene sets and how they relate to development into the main text (figure added as extended data)

The claim to have shown "the first non-invasive window into maternal-fetal development from a maternal liquid biopsy sample" and the comparison with breast cancer liquid biopsy is overstated based on the data and analyses presented for this part of the paper.

Comment #4:
We have removed this from the main text

As one of three main foci of the paper is the claim that cfRNA can reflect fetal development via organ-specific transcripts, functional analysis (e.g. with GO) to show how sets of genes that increase and decrease across gestation provide insight into fetal biology is within the scope of the paper. Currently this part of the manuscript could be improved with more substance.

Comment #5:
We appreciate the feedback and have added analyses on pregnancy related GO categories. In particular, we see highly significant ($\alpha < 0.01$) changes over gestational age for 7 pregnancy-related endocrine signatures (new supplementary figure), this includes drop in gonadotropin secretion and increase in response to estrogen. For functional gene sets that refer to organ development the best proxy is the fetal gene sets used in our primary analyses no GO category (or similar) exists for this.

We have added gene ontology analyses as an addition to the gene set analyses, key findings in main text and data in extended data figure

A minor comment about this aspect of the paper is that Figure 2i-l needs to explain the meaning of the colors used in these panels.

Comment #6:
Thank you for catching this error. We have added a legend explaining the colors.

2. The authors have presented a compelling argument for why a test with PPV in the range of 32% has utility. Assuming that this test is appropriately robust and useful for detecting preeclampsia, the manuscript still needs to detail a more compelling rationale for how detecting preeclampsia risk up to 14.5 weeks in advance would change management or disease course. A basic tenet of screening that a positive screen offers the potential to change the disease trajectory or outcome. The authors themselves state the rationale for earlier diagnosis as identifying those at risk early in pregnancy when there is a "window for preventive strategies." It is not clear that a positive second trimester screen for preeclampsia has that utility. The rationale offered in the response to reviewer comments (to start low dose aspirin and home BP monitoring) is somewhat thin. Low-dose aspirin started much after 12 weeks and certainly after 20 weeks is unlikely to alter the trajectory of preeclampsia, based on biological plausibility of how it might work to impact trophoblast

invasion/placental function. While the mechanisms of LDA for preeclampsia prevention remain unclear, it does seem clear that starting it after 20 weeks for this purpose has little impact. Figure 3a suggests the majority of samples were drawn around 18-20 weeks and with another peak around 24-25 weeks, both of which seem late to start LDA to impact placental function.

Comment #7:
We agree that LDA is documented to help up to 20 weeks of gestation. Thus, some individuals identified by this liquid biopsy would be eligible and likely to benefit from LDA prior to 20 weeks. The American College of Obstetrics and Gynecology (ACOG) and the Society of Maternal Fetal Medicine (SMFM) recommend that use of low dose aspirin should be initiated between 12 and 28 weeks of gestation for those women who are high risk for preeclampsia based on a prior history of the disease.

While this intervention appears most beneficial if started in the 2nd trimester, there are medical interventions currently available for women identified as at risk beyond 20 weeks in pregnancy. These available clinical strategies could improve maternal and/or neonatal outcomes. As approximately 1/3rd of women diagnosed with preeclampsia will have disease that mandates a preterm delivery, the use of antenatal steroids for the mother to decrease neonatal morbidity should not be underestimated. While administration of antenatal steroids has clear benefit for the preterm neonate, providers lack the necessary tools to identify pregnancies at risk and thus fail to provide this proven intervention in an optimal time period in over 60% of individuals who would benefit (Levin et al, 2016, BJOG).

Further, recent studies have demonstrated a positive effect of metformin given to women diagnosed with preeclampsia before 32 weeks (Syngelaki et al, 2021, NEJM). This study and other similar ones suggest that these interventions might also benefit individuals identified as high risk.

Perhaps most importantly, the development of these therapeutics demonstrates that the expectation of pharmacologic innovation in the treatment of preeclampsia is a realistic expectation. Given the multiple therapeutics presently under development with several in phase III clinical trials (metformin and pravastatin), having a predictive test that will stratify those at higher risk for preeclampsia, will not only accelerate therapeutic innovation but also ensure the more focused clinical care delivery.

In addition, if home BP monitoring is the intervention after the screening test, some evidence demonstrating efficacy of home BP monitoring in altering the trajectory of preeclampsia and/or improving maternal outcomes in cohorts at high risk for preeclampsia should be offered in the manuscript's discussion to frame why the screening test with 14 + week lead time will be impactful in translation/why such a test is needed.

Comment #8:
Home blood pressure monitoring is both feasible and, in some ways, preferable as a means to reduce the burdens on the hospital system. A few studies are published demonstrating the utility of home blood pressure monitoring, e.g. Perry et al, 2018, Ultrasound Obstet Gynecol; Hauspurg et al, 2019, Obstet Gynecol.

The ability of the test to predict preeclampsia in the first trimester might lend some biological utility, as the first trimester is a time when starting LDA actually might alter disease course, but the authors state there aren't enough patients in the cohort with first trimester blood draw who had preeclampsia to assess the utility of their predictive test in this setting. To truly demonstrate why this paper represents a "paradigm shift" in preeclampsia prediction, as the authors state in response to reviewer comments, more rationale/explanation must be provided as to why/how such a test will alter disease trajectory or maternal outcomes.

Comment #9:
Today, proactive and personalized care is made available to those women labeled as high risk for preeclampsia based on their obstetric history – which only represents about 15% of all new preeclampsia cases – and there is no reliable way to predict preeclampsia in advance of symptoms. We believe the reason this work represents a "paradigm shift" is that is enables for the first time, a proactive and personalized care approach to be made available to the vast majority of those who would develop preeclampsia. It brings to bear focused clinical care delivery using: 1) the current armamentarium of LDA, home-blood pressure cuff monitoring, and optimal timing for antenatal steroids, as well as 2) identifying a high-risk group to accelerate development of new therapeutics such as metformin, pravastatin and other entities. In the absence of a reliable way to identify who is at risk, we have seen how such development has stagnated and left pregnancy care several decades behind most other areas of medicine.

One use of the test in real-world settings could be to delineate preeclampsia from an exacerbation of chronic hypertension, as the authors describe, but this is not using the test to “predict” preeclampsia, which is the main focus of the paper.

Comment #10:
In our design we test the preeclampsia group against all other labels, this includes chronic hypertensive women. The strength of this design is that our test is closer to real-world settings in that we predict/isolate preeclampsia in a mixed background, this includes delineating from chronic hypertensive women, which is a growing subpopulation. As the obstetrical treatment for chronic hypertension is very different than that for preeclampsia, this function would be highly beneficial.

3. Similarly, more framing of how the advance of dating a pregnancy to the same accuracy as second trimester ultrasound is practice-altering or field-altering would be informative and strengthen the paper. Do the authors feel this would be potentially used in low-resource settings? In settings with limited prenatal care? A brief statement highlighting the relevance would be useful.

Comment #11:
A precise estimate of gestational age can have utility in several settings. CDC estimates that 165,000 women start prenatal care after the 24th week of pregnancy, here a cfRNA-based test could enable more accurate dating and ensure appropriate prenatal care is administered. In areas without access to a trained sonographer, a blood sample shipped to a central lab could offer an alternative and simpler solution. With further development a PCR based test would benefit families in lower resource settings.

4. There is still insufficient detail provided in the main text about how the case-control study was conducted in cohorts A and E. How/based on what criteria were the 72 “cases” of preeclampsia matched to 452 “non-cases”? For example, the rationale for matching gestational hypertension as a “non-case”/control is unclear. Gestational hypertension has been increasingly recognized as on the spectrum of preeclampsia and has similar implications for maternal health and pregnancy management, so wouldn’t be an appropriate “control” against which to match preeclampsia samples for a prediction model. It might be worthwhile to examine what happens if these 19 cases from cohort E were grouped with the preeclampsia cases instead, as both are thought to be a “placental disorder” in contrast to chronic hypertension (e.g. page 8 line 253). More detail should be provided about the “case-control” design here as preeclampsia prediction appears to be the main feature of the paper as currently written.

Comment #12:
We will clarify further in the text how samples were selected. We strived to ensure that the preeclampsia classifier presented, is representative of and applicable to a real-world setting. As such, the preeclampsia cases were matched 2:1 on gestational age at blood draw, race, BMI and maternal age. The non-case population was further expanded to include available samples from the gestational age analyses as well as samples with spontaneous preterm delivery.

Since our non-case population is selected broadly, it includes samples from pregnancies with other complications such as spontaneous preterm birth, chronic or gestational hypertension. This should make the classifier more robust to real world scenarios, over a classifier that separates preeclampsia from normotensive healthy women. Our classifier was built to predict preeclampsia (AUC=0.82). When removing gestational hypertensive women from the non-case population our performance remains at AUC=0.82, indicating that gestational hypertensive women behave as any other non-case sample. If we merge gestational hypertensive and preeclampsia individuals and treat them all as cases, our performance yields an AUC=0.76; this slightly lower performance indicates that our classifier separates preeclampsia from gestational hypertension. This finding is actually clinically valuable as women with gestational hypertension have less severe consequences for that pregnancy for both mother and fetus, than those diagnosed with preeclampsia, and so our classifier identifies the more severe condition.

We have expanded our description of the population used for the preeclampsia study in the supplementary material and edited the main text to remove confusion around how matching was done.

6. The demonstration that the biology is independent of race in preeclampsia is useful and the authors should be commended for framing their results in this manner.

Referee #2 (Remarks to the Author):

The authors have revised their manuscript and thoughtfully addressed reviewer comments raised by myself and others. Below, I have a few additional questions that were not clarified for some specific comments:

1. The authors addressed Reviewer 2 comment 18 to add the timepoint of sample collection, but do not address the original comment: "This manuscript is using a number of different cohorts that are from very different parts of the world, and with data collected at very different timepoints, and with very different goals. It would be helpful to clarify this in the manuscript text, and in table one to clarify the dates of cohort collection and/or if collection is still ongoing. This is relevant because some of these studies span from 1998 to the present day, and there are a number of fundamental changes that have occurred in prenatal care and the treatment of gestational diseases within this timespan." I do not see the date range when the samples were included (For example, the POUCH study was collected from 1998-2004, but the other studies do not have this information listed) in either Table 1 or Supplemental Table 1. This is also not mentioned in the manuscript text as a concern.

Comment #13:
We have added ranges for each cohort for when the samples in the study were collected. For the gestational age analyses, we are only using basic information such as ultrasound estimated gestational age, alongside clinical factors, neither of these are expected to have changed materially over the period the samples were collected. For the two cohorts involved in the preeclampsia analyses, samples were adjudicated after the 2013 ACOG guidelines, so outcomes are fully comparable.

2. I am still confused about the presentation of race/ethnicity in table 1, where Hispanic/non Hispanic (ethnicity) should be separated from black, Asian, white (Race). Otherwise, it looks like the authors addressed this.

Comment #14:
For our description of race and ethnicity we are following the latest guidelines just published in JAMA (Flanagin et al, JAMA, Aug 2021), here it is recommended to report race and ethnicity together and not separate them out. To be fully compliant with these new guidelines we have also added description of what is captured under each our labels, this has been added to the supplementary text.

3. In response to reviewer comments (from myself and others) about the challenges of presenting the data from only cohort H in the model, the authors add panels I-L to demonstrate signal across all cohorts with longitudinal data. However, there are only 4 lines here and it is not clear what color corresponds to which cohort. Can the authors clarify this picture.

Comment #15:
Please refer to comment #6, this has been fixed in the revised manuscript

Referee #3 (Remarks to the Author):

The authors have adequately addressed my prior comments. I have no further comments on the manuscript and study.

Reviewer Reports on the Second Revision:

Referee #1 (Remarks to the Author):

The authors have adequately addressed my prior comments. I have no further comments on the paper.

Referee #2 (Remarks to the Author):

The authors of this manuscript have addressed all reviewer concerns.
